# Risk of advanced neoplasia after removal of colorectal adenomas with high-grade dysplasia

**DOI:** 10.1007/s00464-024-10898-5

**Published:** 2024-05-28

**Authors:** Degao He, Kai Wang, Yanhong Zhang, Xuefei Jiang, Hao Chen, Junguo Chen, Danlin Liu, Guanman Li, Jiancong Hu, Xiaosheng He

**Affiliations:** 1 Department of Anorectal Surgery, Shenzhen Longhua District Central Hospital, Guanlan Avenue 187, Shenzhen, 518100 Guangdong China; 2 Department of Anaesthesia, The Sixth Affiliated Hospital, Sun Yat-sen University, 26 Yuancun Er Heng Road, Guangzhou, 510655 Guangdong China; 3 Department of General Surgery (Colorectal Surgery), The Sixth Affiliated Hospital, Sun Yat-sen University, 26 Yuancun Er Heng Road, Guangzhou, 510655 Guangdong China; 4 Department of General Surgery (Institute of Gastroenterology), The Sixth Affiliated Hospital, Sun Yat-sen University, 26 Yuancun Er Heng Road, Guangzhou, 510655 Guangdong China; 5 Department of General Surgery (Endoscopic Surgery), The Sixth Affiliated Hospital, Sun Yat-sen University, 26 Yuancun Er Heng Road, Guangzhou, 510655 Guangdong China; 6 Guangdong Provincial Key Laboratory of Colorectal and Pelvic Floor Diseases, Guangdong Institute of Gastroenterology, The Sixth Affiliated Hospital, Sun Yat-sen University, 26 Yuancun Er Heng Road, Guangzhou, 510655 Guangdong China; 7 Biomedical Innovation Center, The Sixth Affiliated Hospital, Sun Yat-sen University, 26 Yuancun Er Heng Road, Guangzhou, 510655 Guangdong China

**Keywords:** Colorectal adenoma, High-grade dysplasia, Advanced neoplasia, Colorectal cancer, Colonoscopy

## Abstract

**Background:**

Many studies reported the presence of adenomas with high-grade dysplasia (HGD) at index colonoscopy increased the incidence of advanced neoplasia (AN) and colorectal cancer (CRC) following. However, the conclusion remains obscure due to lack of studies on the specific population of adenomas with HGD. This study aimed to assess the long-term risk of AN and CRC after removal of adenomas with HGD.

**Methods:**

A total of 814 patients who underwent adenomas with HGD removal between 2010 and 2019 were retrospectively analyzed. The outcomes were the incidences of AN and CRC during surveillance colonoscopy. Cox proportional hazards models were utilized to identify risk factors associated with AN and CRC.

**Results:**

During more than 2000 person-years of follow-up, we found that AN and CRC incidence densities were 44.3 and 4.4 per 1000 person-years, respectively. The 10-year cumulative incidence of AN and CRC were 39.1% and 5.5%, respectively. In the multivariate model, synchronous low-risk polyps (HR 1.80, 95% CI 1.10–2.93) and synchronous high-risk polyps (HR 3.99, 95% CI 2.37–6.72) were risk factors for AN, whereas participation in surveillance colonoscopy visits (HR 0.56, 95% CI 0.36–0.88 for 1 visit; HR 0.10, 95% CI 0.06–0.19 for ≥ 2 visits) were associated with decreased AN incidence. Additionally, elevated baseline carcinoembryonic antigen (CEA) level (HR 10.19, 95% CI 1.77–58.59) was a risk factor for CRC, while participation in ≥ 2 surveillance colonoscopy visits (HR 0.11, 95% CI 0.02–0.56) were associated with decreased CRC incidence. Interestingly, for 11 patients who developed CRC after removal of adenomas with HGD, immunohistochemistry revealed that 8 cases (73%) were deficient mismatch repair CRCs.

**Conclusions:**

Patients who have undergone adenoma with HGD removal are at higher risk of developing AN and CRC, while surveillance colonoscopy can reduce the risk. Patients with synchronous polyps, or with elevated baseline CEA level are considered high-risk populations and require more frequent surveillance.

**Supplementary Information:**

The online version contains supplementary material available at 10.1007/s00464-024-10898-5.

Colorectal cancer (CRC) is the third most common cancer and the second leading cause of cancer-related death worldwide [1]. High-quality studies have demonstrated that endoscopic screening for CRC significantly reduces CRC incidence and mortality rates [2–5]. The reduction in incidence is attributed to the removal of premalignant polyps (PMPs), including adenomas and serrated polyps [6]. However, as polyps can recur, patients with a history of PMPs are at higher risk of developing metachronous polyps and CRCs [7, 8]. As per current national guidelines, these patients require frequent surveillance colonoscopies to prevent subsequent cancers [9–11]. With the implementation of CRC screening programs in many countries, there has been an increase in the number of individuals with PMP findings. As individuals with PMPs tend to undergo surveillance colonoscopy more frequently, this may lead to unnecessary risks such as adverse events or psychological distress, as well as increased healthcare costs. Therefore, it is important to target surveillance colonoscopies at individuals who are most likely to benefit from them.

According to current national guidelines, the post-polypectomy surveillance interval is determined by the number, size, and histology of the detected polyps. For instance, the 2020 UK guidelines recommend endoscopic surveillance every 3 years for high-risk individuals with two or more PMPs, of which at least one is considered “advanced” [e.g., adenoma ≥ 10 mm or with high-grade dysplasia (HGD); serrated polyp ≥ 10 mm or with dysplasia]; five or more PMPs; or at least one ≥ 20 mm non-pedunculated PMP [10]. The 2020 US guidelines use similar polyp characteristics to identify individuals who require intensive surveillance (e.g., PMP ≥ 10 mm or with HGD, five or more PMPs) [11]. HGD is a critical step in the progression of adenoma to CRC [12]. Both the UK and US guidelines consider adenomas with HGD as high-risk PMPs. Most previous studies have demonstrated that the presence of HGD at index colonoscopy significantly increases the incidence of advanced neoplasia (AN) [13–17]. However, some studies found no significant association between HGD at index and AN risk [18–20], and even one study suggested a reduction in HGD-related AN risk [21]. Could this discrepancy be due to some factors within the population of adenomas with HGD? Unfortunately, previous studies only treated HGD as a baseline characteristic, and specific studies on the population of adenomas with HGD is lacking. Moreover, the incidence of metachronous AN or CRC after removal of adenomas with HGD remains unclear.

To address these knowledge gaps, we conducted a study specifically focused on a population of adenomas with HGD to investigate their long-term incidence of AN and CRC. We explored whether baseline characteristics and surveillance colonoscopy influence the incidence of AN and CRC and further subdivided the risk stratification in this population, thereby improving the scientific basis for postoperative surveillance strategies.

## Materials and methods

### Study design and patients

We designed a retrospective cohort study using data from patients who underwent polypectomy at the Six Affiliated Hospital of Sun Yat-sen University in Guangzhou, China. We searched the hospital pathology database with the keyword “high-grade dysplasia” to identified patients with a pathological diagnosis of adenoma with HGD between January 2010 and December 2019. Patients with a previous diagnosis of adenoma with HGD before January 2010 were not considered. The first diagnosis of adenoma with HGD was defined as the “baseline”. A colonoscopy performed after the baseline was considered a surveillance colonoscopy. All colonoscopies were performed by experienced endoscopists. Each endoscopist has completed standardized training in the field of endoscopy, and is proficient in the operating procedures and techniques of colonoscopy. They are capable of independently handling any unexpected events that may occur during the procedure, and accurately documenting the findings in the colonoscopy report upon completion. Lesions detected within 6 months after the baseline were classified as “baseline lesions” that might be previously missed during the baseline colonoscopy. This practice was designed to reduce the impact of missed lesions on the final results and to help more accurately estimate the risk of developing advanced neoplasia postoperatively.

To be included, patients were required to have at least one adenoma with HGD at the baseline colonoscopy. We excluded patients diagnosed with CRC at or before the baseline examination, as well as those with inflammatory bowel disease, familial adenomatous polyposis, Lynch syndrome, Peutz-Jeghers syndrome, or without necessary baseline data. Furthermore, patients who did not undergo colonoscopy after the baseline were excluded. The study was approved by the Ethics Review Committee of the Sixth Affiliated Hospital of Sun Yat-sen University.

### Data collection

We conducted a thorough search of the hospital's medical notes, endoscopy databases, and pathology databases to collect data, including patients' age and sex, as well as the size, number, location, histology and operative modality of lesions detected at baseline colonoscopy. We also recorded the quality of the baseline colonoscopy and baseline serum carcinoembryonic antigen (CEA) concentrations. For follow-up data, we collected information about each surveillance colonoscopy, including the date of examination and detailed characteristics of any lesions detected such as their size, number, location, and histology. The histological features of the lesions were evaluated by specialized gastrointestinal pathologists according to the colorectal neoplasia classification of the World Health Organization recommendations.

### Outcome measures

The primary outcome measure was the incidence of AN during surveillance colonoscopy. AN was defined in accordance with current guidelines as CRC, advanced adenomas (≥ 10 mm, or containing tubulovillous/villous histology or HGD), and advanced serrated polyps (≥ 10 mm or containing any grade of dysplasia) [10, 11]. Incidence density rate (number of cases per 1000 patient-years) and cumulative incidence were used to express the incidence of AN. The secondary outcome measure was the incidence of CRC, also expressed as incidence density rate and cumulative incidence. We collected all surveillance colonoscopies performed prior to November 30, 2022. If there were outcome events (AN or CRC) present, these were considered as the follow-up endpoints. If no outcome events occurred, the date of the last colonoscopy represented the endpoint of follow-up time. Prior to the statistical analysis, we excluded any CRCs or adenomas with HGD that developed in the same colonic segment within one year after the baseline examination, considering these lesions as incomplete resection of the adenomas with HGD at baseline.

### Definitions

In this study, the standardized abstraction of data from endoscopic and pathologic reports included information on the size, number, location and histology of lesions and the quality of colonoscopy. For multiple lesions, the size, location, and histology were classified according to the largest, the most proximal, and the most advanced lesion. The location of the lesion was classified as either distal or proximal to the splenic flexure. Bowel cleansing and cecal intubation were noted in the endoscopic report, and colonoscopies were designated as inadequate if bowel cleansing was rated “poor” or “very poor” according to the Aronchick scale [22], or if cecal intubation could not be achieved.

For polyps identified during colonoscopy, treatment methods were typically determined by professional endoscopists after considering the polyp's shape, size, number, and patient's age. For microscopic polyps (< 5 mm) and small polyps (5–10 mm), electrocoagulation, biopsy forceps, and endoscopic snare polypectomy were commonly used, while large polyps (> 10 mm) were usually treated with endoscopic mucosal resection, and endoscopic submucosal dissection. In addition, some large polyps located in the lower rectal were treated with transanal local excision. In most cases, multiple polyps identified during colonoscopy were removed simultaneously. However, if the patient's bowel preparation was insufficient or the patient could not tolerate a long operation time, and some of the detected polyps could not be removed simultaneously, a second colonoscopy would be scheduled within 1 month to remove the remaining polyps. Specifically, for polyps smaller than 5 mm located in the distal colon and rectum, polyps between 3 and 5 mm were typically removed by forceps biopsy, and those less than 3 mm were treated with electrocautery. In our cohort, segmental bowel resection was performed in some patients when preoperative assessment of submucosal infiltration of adenomas with HGD was inconclusive. Endoscopic procedures such as biopsy forceps, endoscopic snare polypectomy, endoscopic mucosal resection, and endoscopic submucosal dissection, as well as transanal local excision, were classified as local resection. In addition to adenomas with HGD, some patients had synchronous PMPs at baseline colonoscopy. Synchronous PMPs were categorized as either high-risk polyps (including tubulovillous adenomas, villous adenomas, sessile serrated polyps, traditional serrated adenomas, polyps ≥ 10 mm in size, and polyps ≥ 5 in number) or low-risk polyps (polyps < 10 mm in size and < 5 in number and containing exclusively tubular/hyperplastic histology). These classifications applied to PMPs with the exception of adenomas with HGD. Furthermore, the normal range of serum CEA level was 0–4.9 ng/mL. CEA level ≥ 5 ng/mL was defined as elevated.

We further assess the impact of postoperative surveillance colonoscopy on the incidence of AN and CRC. Patients were categorized into three groups based on the number of surveillance visits: 0 surveillance visit (no colonoscopy performed between baseline colonoscopy and last colonoscopy), 1 surveillance visit (one colonoscopy performed between baseline colonoscopy and last colonoscopy), and ≥ 2 surveillance visits (at least two colonoscopies performed between baseline colonoscopy and last colonoscopy).

### Statistical analysis

We performed the following analysis for the whole cohort. We utilized univariable and multivariable Cox proportional hazards models to estimate HRs with 95% CIs to examine the effects of baseline characteristics and surveillance visits on the incidence of AN or CRC. Backward stepwise selection was employed to retain variables with *P* values < 0.05 in likelihood ratio tests within the multivariate model to identify independent AN or CRC risk factors. We performed Kaplan–Meier analyses to estimate cumulative AN or CRC incidence at 5 and 10 years with 95% CIs. Differences in cumulative incidence were tested using the log-rank test. All statistical analyses were two tailed and *P* values < 0.05 were considered significant. All statistical analyses were performed using Stata/SE V.15.1 [23].

### Sensitivity analyses

In present study, the quality of the baseline colonoscopy was considered a baseline characteristic. Previous studies have shown that inadequate baseline colonoscopy is significantly associated with an increased risk of CRC [24, 25]. To eliminate this effect, we performed a sensitivity analysis that only included patients with adequate baseline colonoscopy. In another sensitivity analysis, we did not exclude patients who developed CRC or adenoma with HGD in the same intestinal segment within one year after baseline. Because there is no definitive evidence supporting their association with baseline lesions, and excluding them could potentially underestimate the risk of AN and CRC.

## Results

### Population characteristics and follow-up outcomes

**Fig. 1:**
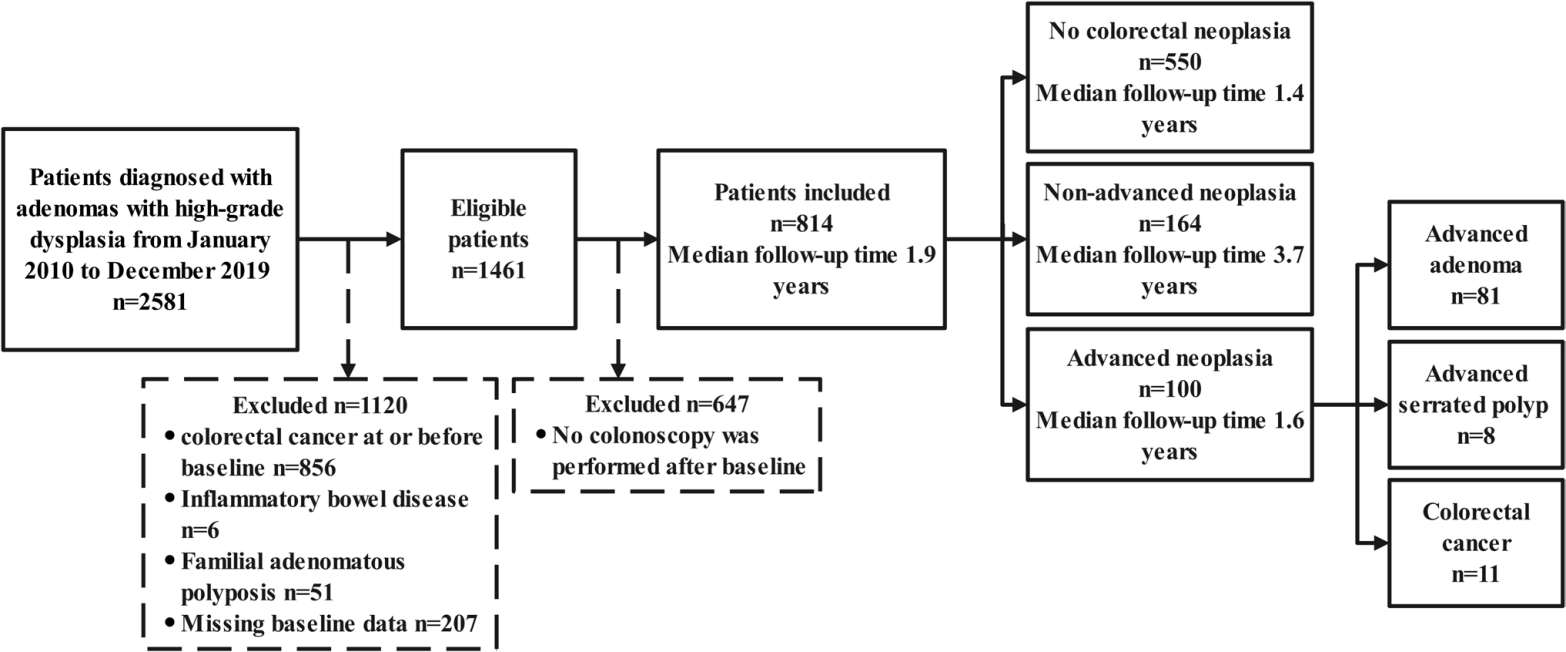
Flow chart of the study population and follow-up findings

We identified 2581 patients who could be considered for inclusion in our study. Of these, we excluded 856 with CRC at or before baseline; 6 with inflammatory bowel disease; 51 with familial adenomatous polyposis; 207 without required baseline information; 647 who did not undergo colonoscopy after baseline. Finally, 814 patients were included (Fig. 1). Among 814 eligible patients, 306 (37.6%) were female, and the mean age was 55 years (range 20–86 years). The median follow-up time was 1.9 years (IQR 3.9–0.7), while the median time from baseline to first surveillance was 0.5 years (IQR 1.1–0.3). During 2168.5 person-years of follow-up, we identified 100 ANs, including 81 advanced adenomas, 8 advanced serrated polyps, and 11 CRCs (Fig. 1; Supplementary Table S1).

### Incidence of AN

Prior to this statistical analysis, we excluded 4 cases of AN (3 adenomas with HGD and 1 CRC) as we assumed they had developed from incomplete resection of the adenomas with HGD at baseline. The remaining 810 patients were followed for 2165.9 person-years and 96 ANs were diagnosed, giving an incidence rate of 44.3 per 1000 person-years. Univariate analysis showed that age ≥ 75 years, ≥ 2 adenomas with HGD, and synchronous low-risk polyps or high-risk polyps were significantly associated with increased AN incidence. However, participation in ≥ 2 surveillance visits was significantly associated with decreased AN incidence. All significant variables in univariate analysis (*P* < 0.05) were included in multivariate analysis. In the multivariate model, we determined that synchronous low-risk polyps (HR 1.80, 95% CI 1.10–2.93) or high-risk polyps (HR 3.99, 95% CI 2.37–6.72) were independently associated with increased AN incidence, while participation in ≥ 1 surveillance visits (HR 0.56, 95% CI 0.36–0.88 for 1 visit; HR 0.10, 95% CI 0.06–0.19 for ≥ 2 visits) was independently associated with decreased AN incidence (Table 1).

**Table 1:** Long-term incidence of advanced neoplasia by baseline characteristics and number of surveillance visits

	*n* (%)	Number of polyps identified at baseline	No of person-years	No of ANs	Incidence rate per 1000 person-years	Univariable HR (95% CI)	*P* value^a^	Multivariable HR (95% CI)	*P* value^a^
Total	810 (100)	1903	2165.9	96	44.3				
Sex									
Women	303 (37.4)	596	798.4	32	40.1	1 (Ref)			
Men	507 (62.6)	1307	1367.5	64	46.8	1.17 (0.76–1.78)	0.481		
Age at baseline (years)									
< 55	364 (45.9)	712	1132.1	41	36.2	1 (Ref)		1 (Ref)	
55–64	242 (29.9)	648	586.9	25	42.6	1.20 (0.73–1.98)	0.476	1.04 (0.62–1.73)	0.896
65–74	154 (19.0)	385	341.8	20	58.5	1.68 (0.98–2.88)	0.061	1.27 (0.73–2.21)	0.402
≥ 75	50 (6.2)	158	105.1	10	95.1	2.72 (1.36–5.47)	0.005	1.44 (0.70–2.98)	0.327
Location of adenomas with HGD^b^									
Only distal	615 (75.9)	1361	1633.7	68	41.6	1 (Ref)			
Any proximal	195 (24.1)	542	532.2	28	52.6	1.26 (0.81–1.96)	0.300		
Number of adenomas with HGD									
1	726 (89.6)	1559	1925.5	78	40.5	1 (Ref)		1 (Ref)	
≥ 2	84 (10.4)	344	240.4	18	74.9	1.83 (1.09–3.05)	0.022	1.55 (0.92–2.59)	0.100
Size of adenomas with HGD^c^									
< 10 mm	109 (13.5)	265	312.4	11	35.2	1 (Ref)			
10–19 mm	304 (37.5)	758	832.0	37	44.5	1.26 (0.64–2.47)	0.505		
≥ 20 mm	397 (49.0)	880	1021.5	48	47.0	1.36 (0.71–2.62)	0.357		
Synchronous PMPs^d^									
No	387 (47.8)	418	1067.1	29	27.2	1 (Ref)		1 (Ref)	
Low-risk polyps	304 (37.5)	876	763.9	37	48.4	1.78 (1.09–2.90)	0.020	1.80 (1.10–2.93)	0.019
High-risk polyps	119 (14.7)	609	334.9	30	89.6	3.26 (1.96–5.43)	< 0.001	3.99 (2.37–6.72)	< 0.001
Resection range^e^									
Segmental bowel resection	114 (14.1)	215	278.2	10	35.9	1 (Ref)			
Local resection	696 (85.9)	1688	1887.7	86	45.6	1.25 (0.65–2.41)	0.503		
Quality of baseline colonoscopy^f^									
Adequate	742 (91.6)	1706	1992.7	90	45.2	1 (Ref)			
Inadequate	68 (8.4)	197	173.2	6	34.6	0.79 (0.34–1.80)	0.571		
Baseline serum CEA level									
0–4.9 ng/mL	554 (68.4)	1292	1408.6	67	47.6	1 (Ref)			
≥ 5 ng/mL	44 (5.4)	137	75.4	7	92.8	2.02 (0.93–4.42)	0.077		
NA	212 (26.2)	474	681.9	22	32.3	0.67 (0.41–1.08)	0.103		
Number of surveillance visits									
0	392 (48.4)	889	577.8	50	86.5	1 (Ref)		1 (Ref)	
1	198 (24.4)	480	529.5	33	62.3	0.65 (0.42–1.02)	0.060	0.56 (0.36–0.88)	0.013
≥ 2	220 (27.2)	534	1058.5	13	12.3	0.12 (0.06–0.22)	< 0.001	0.10 (0.06–0.19)	< 0.001

*AN* advanced neoplasia, *HR* hazard ratio, *CI* confidence interval, *HGD* high-grade dysplasia, *PMP* premalignant polyp, *CEA* carcinoembryonic antigen, *NA* not available

^a^*P* values were calculated with the likelihood ratio test. Differences with *P* < 0.05 were considered statistically significant

^b^Distal was defined as distal to the splenic flexure. Proximal was defined as proximal to the splenic flexure

^c^Size was defined according to the largest adenomas with HGD seen at baseline

^d^Low-risk polyps were defined as polyps < 10 mm in size and < 5 in number and containing exclusively tubular/hyperplastic histology. High-risk polyps included tubulovillous adenomas, villous adenomas, sessile serrated polyps, traditional serrated adenomas, polyps ≥ 10 mm in size and polyps ≥ 5 in number

^e^Local resection included endoscopic resection (e.g., cold/hot biopsy forceps, endoscopic snare polypectomy, endoscopic mucosal resection, endoscopic submucosal dissection) and transanal local excision

^f^The baseline colonoscopy was inadequate if bowel cleansing was rated “poor” or “very poor”, or if cecal intubation could not be achieved

**Fig. 2:**
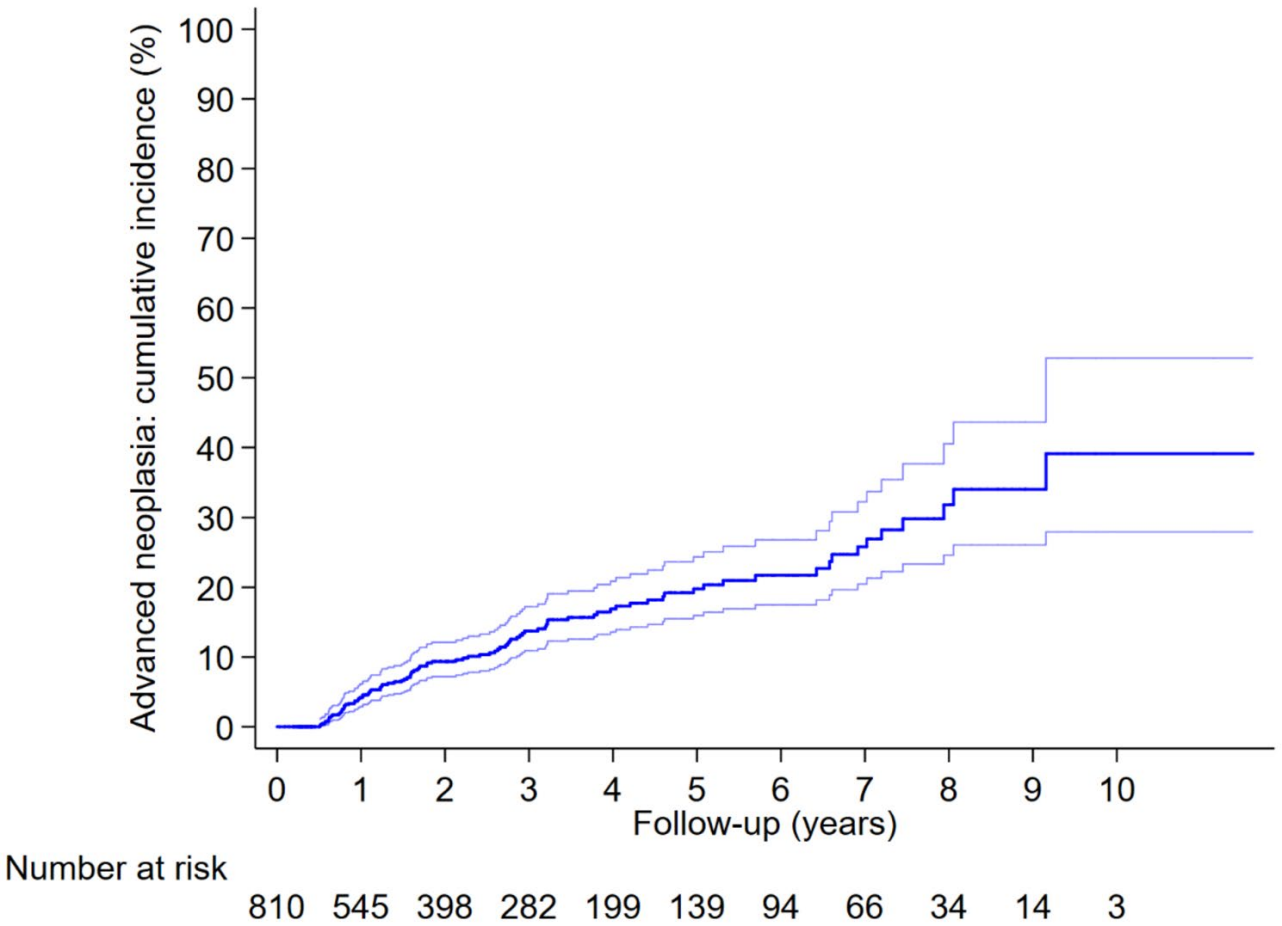
The cumulative incidence and its 95% confidence interval curve of advanced neoplasia in the whole cohort. The cumulative incidence was estimated by Kaplan–Meier analysis

In the whole cohort, the cumulative AN incidence at 5 and 10 years was 19.8% (95% CI 16.0–24.4%) and 39.1% (95% CI 27.9–52.8%), respectively. Age ≥ 75 years, ≥ 2 adenomas with HGD, synchronous low or high-risk polyps, and elevated baseline CEA level significantly increased the cumulative AN incidence. In contrast, participation in ≥ 1 surveillance visits significantly reduced the cumulative AN incidence (Fig. 2; Table 2).

**Table 2:** Cumulative incidence of advanced neoplasia after removal of adenoma with HGD

	*n* (%)	No of person-years	No of ANs	Incidence rate per 1000 person-years	5-year cumulative incidence (95% CI)^a^	10-year cumulative incidence (95% CI)^a^	*P* value^b^
Total	810 (100)	2165.9	96	44.3	19.8% (16.0–24.4)	39.1% (27.9–52.8)	
Sex							0.480
Women	303 (37.4)	798.4	32	40.1	19.4% (13.3–27.7)	31.6% (20.3–47.1)	
Men	507 (62.6)	1367.5	64	46.8	20.0% (15.4–25.8)	44.9% (28.1–65.9)	
Age at baseline (years)							0.003
< 55	364 (45.9)	1132.1	41	36.2	15.8% (11.2–21.9)	33.2% (19.9–52.0)	
55–64	242 (29.9)	586.9	25	42.6	18.2% (11.8–27.5)	38.0% (22.1–59.9)	
65–74	154 (19.0)	341.8	20	58.5	24.7% (15.7–37.7)	–	
≥ 75	50 (6.2)	105.1	10	95.1	46.7% (26.9–71.7)	–	
Location of adenomas with HGD^c^							0.299
Only distal	615 (75.9)	1633.7	68	41.6	18.7% (14.5–24.0)	42.3% (28.0–60.2)	
Any proximal	195 (24.1)	532.2	28	52.6	22.8% (15.5–32.9)	30.8% (19.5–46.6)	
Number of adenomas with HGD							0.020
1	726 (89.6)	1925.5	78	40.5	17.9% (14.1–22.5)	34.4% (22.8–49.6)	
≥ 2	84 (10.4)	240.4	18	74.9	32.1% (19.6–49.6)	–	
Size of adenomas with HGD^d^							0.374
< 10 mm	109 (13.5)	312.4	11	35.2	14.9% (7.9–27.2)	28.2% (14.0–51.7)	
10–19 mm	304 (37.5)	832.0	37	44.5	18.8% (13.0–26.7)	–	
≥ 20 mm	397 (49.0)	1021.5	48	47.0	22.1% (16.4–29.3)	42.4% (25.4–64.6)	
Synchronous PMPs^e^							< 0.001
No	387 (47.8)	1067.1	29	27.2	11.8% (7.8–17.8)	35.7% (16.7–65.6)	
Low-risk polyps	304 (37.5)	763.9	37	48.4	20.4% (14.4–28.3)	35.8% (21.8–55.0)	
High-risk polyps	119 (14.7)	334.9	30	89.6	38.7% (27.6–52.4)	–	
Resection range^f^							0.502
Segmental bowel resection	114 (14.1)	278.2	10	35.9	13.7% (6.9–26.2)	–	
Local resection	696 (85.9)	1887.7	86	45.6	20.6% (16.4–25.6)	36.5% (27.6–47.4)	
Quality of baseline colonoscopy^g^							0.570
Adequate	742 (91.6)	1992.7	90	45.2	20.3% (16.3–25.2)	39.7% (28.0–54.2)	
Inadequate	68 (8.4)	173.2	6	34.6	13.9% (5.9–31.0)	–	
Baseline serum CEA level							0.028
0–4.9 ng/mL	554 (68.4)	1408.6	67	47.6	20.4% (15.8–26.2)	–	
≥ 5 ng/mL	44 (5.4)	75.4	7	92.8	30.1% (13.9–57.6)	–	
NA	212 (26.2)	681.9	22	32.3	16.5% (10.5–25.2)	26.0% (16.1–40.4)	
Number of surveillance visits							< 0.001
0	392 (48.4)	577.8	50	86.5	34.3% (25.8–44.8)	–	
1	198 (24.4)	529.5	33	62.3	28.8% (19.8–40.8)	–	
≥ 2	220 (27.2)	1058.5	13	12.3	6.0% (3.1–11.6)	15.0% (7.6–28.3)	

*AN* advanced neoplasia, *CI* confidence interval, *HGD* high-grade dysplasia, *PMP* premalignant polyp, *CEA* carcinoembryonic antigen, *NA* not available

^a^Cumulative incidence was estimated using the Kaplan–Meier method. Some categories of the specified variable could not be estimated

^b^*P* values were calculated with the log-rank test to compare cumulative incidence among each category of the specified variable. Differences with *P* < 0.05 were considered statistically significant

^c^Distal was defined as distal to the splenic flexure. Proximal was defined as proximal to the splenic flexure

^d^Size was defined according to the largest adenomas with HGD seen at baseline

^e^Low-risk polyps were defined as polyps < 10 mm in size and < 5 in number and containing exclusively tubular/hyperplastic histology. High-risk polyps included tubulovillous adenomas, villous adenomas, sessile serrated polyps, traditional serrated adenomas, polyps ≥ 10 mm in size and polyps ≥ 5 in number

^f^Local resection included endoscopic resection (e.g., cold/hot biopsy forceps, endoscopic snare polypectomy, endoscopic mucosal resection, endoscopic submucosal dissection) and transanal local excision

^g^The baseline colonoscopy was inadequate if bowel cleansing was rated “poor” or “very poor”, or if cecal intubation could not be achieved

### Incidence of CRC

The characteristics of the 11 patients who developed CRC during the follow-up are detailed in Table 3. The cases comprised 8 moderately differentiated adenocarcinomas, 2 mucinous adenocarcinomas, and 1 spindle cell carcinoma. The time to onset of CRC ranged from half a year to 7 years, with an average of 3.4 years. In 8 cases (73%), CRC occurred in the colorectal region distal to the splenic flexure. Immunohistochemistry revealed that 8 cases (73%) belonged to deficient mismatch repair (dMMR) CRCs. Patient No. 11 was excluded from the statistical analysis because it was considered to have developed from incomplete resection of the adenomas with HGD at baseline. Of the remaining 813 patients, 10 developed CRC during a total follow-up of 2269.9 person-years, resulting in an incidence rate of 4.4 per 1000 person-years. In the multivariate model, we found that elevated baseline CEA level (HR 10.19, 95% CI 1.77–58.59) was an independent risk factor for CRC, while participation in ≥ 2 surveillance visits (HR 0.11, 95% CI 0.02–0.56) was independently associated with decreased CRC incidence (Table 4).

**Table 3:** The characteristics of the eleven colorectal cancer patients identified during follow-up

No	Sex	Age at baseline (years)	Location of adenomas with HGD	Resection method	Time from baseline to CRC (years)	Location of CRC	TNM stage	Differentiated degree	Histological Type	MLH1	MSH2	MSH6	PMS2
1	Male	44	Rectum	Endoscopic mucosal resection	5.2	Transverse colon	T1N0M0	Moderately-poorly	Adenocarcinoma	−	+	+	−
2	Female	51	Rectum	Endoscopic snare polypectomy	6.4	Sigmoid colon	T1N0M0	Moderately	Adenocarcinoma	+	+	+	−
3	Female	50	Rectum	Transanal local excision	4.2	Rectum	T3N0M0	Moderately	Adenocarcinoma	+	+	+	+
4	Male	68	Sigmoid colon	Endoscopic snare polypectomy	7.0	Ascending colon	T1N0M0	Moderately	Adenocarcinoma	+	−	+	−
5	Female	74	Rectum	Endoscopic snare polypectomy	3.2	Ascending colon	T1N0M0	Moderately	Adenocarcinoma	−	−	−	−
6	Female	59	Cecum, ascending colon	Endoscopic mucosal resection	5.3	Rectum	T3N0M0	Moderately	Adenocarcinoma	+	−	−	+
7	Female	46	Cecum, ascending colon	Endoscopic mucosal resection	0.9	Sigmoid colon	T2N0M0	–	Mucinous adenocarcinoma	−	+	+	−
8	Male	53	Rectum	Transanal local excision	1.1	Rectum	T2N0M0	Moderately	Adenocarcinoma	+	+	+	+
9	Male	75	Rectum, transverse colon	Endoscopic snare polypectomy	2.8	Rectum	T4N0M0	–	Spindle cell carcinoma	−	−	+	−
10	Male	63	Transverse colon	Endoscopic mucosal resection	0.5	Descending colon	T1N0M0	Moderately	Adenocarcinoma	−	+	+	−
11	Male	68	Rectum	Transanal local excision	0.8	Rectum	T3N0M0	Highly	Mucinous adenocarcinoma	+	+	+	+

*CRC* colorectal cancer, *HGD* high-grade dysplasia

**Table 4:** Long-term incidence of colorectal cancer by baseline characteristics and number of surveillance visits

	*n* (%)	No of person-years	No of CRCs	Incidence rate per 1000 person-years	Univariable HR (95% CI)	*P* value^a^	Multivariable HR (95% CI)	*P* value^a^
Total	813 (100)	2269.9	10	4.4				
Sex								
Women	306 (37.6)	830.8	5	6.0	1 (Ref)			
Men	507 (62.4)	1439.1	5	3.5	0.60 (0.17–2.07)	0.416		
Age at baseline (years)								
< 55	366 (45.0)	1187.5	5	4.2	1 (Ref)			
55–64	242 (29.8)	607.0	2	3.3	0.94 (0.18–4.87)	0.938		
65–74	155 (19.1)	366.1	2	5.5	1.57 (0.30–8.21)	0.593		
≥ 75	50 (6.1)	109.4	1	9.1	3.12 (0.35–27.7)	0.308		
Location of adenomas with HGD^b^								
Only distal	618 (76.0)	1704.7	6	3.5	1 (Ref)			
Any proximal	195 (24.0)	565.2	4	7.1	2.02 (0.57–7.16)	0.277		
Number of adenomas with HGD								
1	729 (89.7)	2014.2	7	3.5	1 (Ref)			
≥ 2	84 (10.3)	255.8	3	11.7	3.50 (0.90–13.56)	0.070		
Size of adenomas with HGD^c^								
< 10 mm	109 (13.4)	327.0	1	3.1	1 (Ref)			
10–19 mm	306 (37.6)	871.6	5	5.7	2.11 (0.24–18.17)	0.498		
≥ 20 mm	398 (49.0)	1071.4	4	3.7	1.29 (0.14–11.58)	0.819		
Synchronous PMPs^d^								
No	388 (47.7)	1120.9	2	1.8	1 (Ref)			
Low-risk polyps	305 (37.5)	790.8	5	6.3	3.84 (0.74–19.81)	0.108		
High-risk polyps	120 (14.8)	358.2	3	8.4	4.73 (0.79–28.35)	0.089		
Resection range^e^								
Segmental bowel resection	114 (14.0)	293.9	0	0	1 (Ref)			
Local resection	699 (86.0)	1976.1	10	5.1	25.33 (0.01–66291.71)	0.421		
Quality of baseline colonoscopy^f^								
Adequate	745 (91.6)	2093.8	9	4.3	1 (Ref)			
Inadequate	68 (8.4)	176.2	1	5.7	1.38 (0.17–10.94)	0.761		
Baseline serum CEA level								
0–4.9 ng/mL	56 (68.4)	1477.6	4	2.7	1 (Ref)		1 (Ref)	
≥ 5 ng/mL	44 (5.4)	88.9	2	22.5	8.98 (1.64–49.28)	0.012	10.19 (1.77–58.59)	0.009
NA	213 (26.2)	703.4	4	5.7	2.02 (0.50–8.08)	0.321	1.74 (0.43–6.98)	0.436
Number of surveillance visits								
0	374 (46.0)	557.2	6	10.8	1 (Ref)		1 (Ref)	
1	197 (24.2)	543.2	2	3.7	0.32 (0.06–1.60)	0.165	0.27 (0.05–1.37)	0.113
≥ 2	242 (29.8)	1169.6	2	1.7	0.11 (0.02–0.57)	0.008	0.11 (0.02–0.56)	0.008

*CRC* colorectal cancer, *HR* hazard ratio, *CI* confidence interval, *HGD* high-grade dysplasia, *PMP* premalignant polyp, *CEA* carcinoembryonic antigen, *NA* not available

^a^*P* values were calculated with the likelihood ratio test. Differences with *P* < 0.05 were considered statistically significant

^b^Distal was defined as distal to the splenic flexure. Proximal was defined as proximal to the splenic flexure

^c^Size was defined according to the largest adenomas with HGD seen at baseline

^d^Low-risk polyps were defined as polyps < 10 mm in size and < 5 in number and containing exclusively tubular/hyperplastic histology. High-risk polyps included tubulovillous adenomas, villous adenomas, sessile serrated polyps, traditional serrated adenomas, polyps ≥ 10 mm in size and polyps ≥ 5 in number

^e^Local resection included endoscopic resection (e.g., cold/hot biopsy forceps, endoscopic snare polypectomy, endoscopic mucosal resection, endoscopic submucosal dissection) and transanal local excision

^f^The baseline colonoscopy was inadequate if bowel cleansing was rated “poor” or “very poor”, or if cecal intubation could not be achieved

**Fig. 3:**
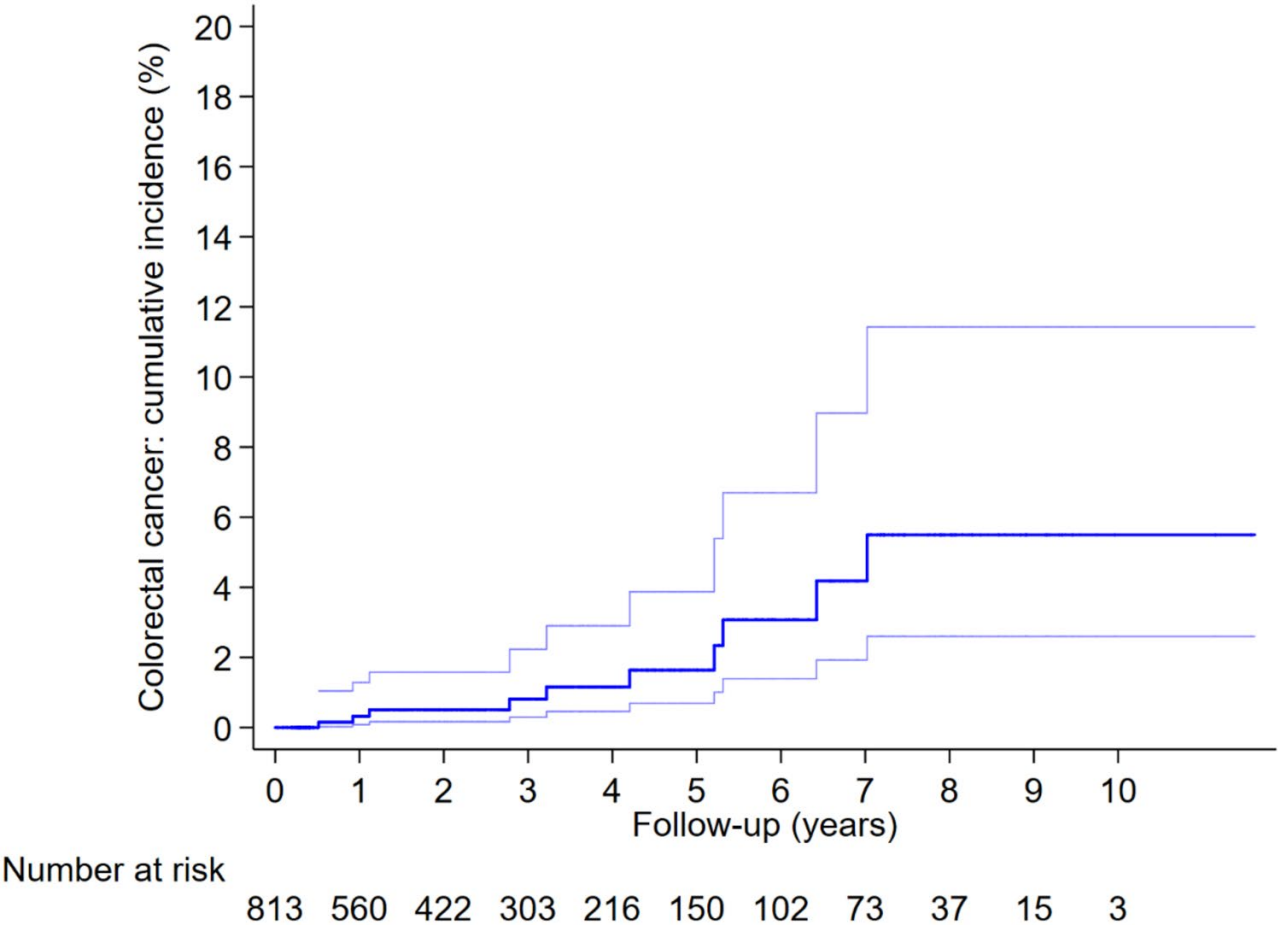
The cumulative incidence and its 95% confidence interval curve of colorectal cancer in the whole cohort. The cumulative incidence was estimated by Kaplan–Meier analysis

In the whole cohort, the cumulative CRC incidence at 5 and 10 years was 1.6% (95% CI 0.7–3.9%) and 5.5% (95% CI 2.6–11.4%), respectively. Elevated baseline CEA level significantly increased the cumulative CRC incidence. In contrast, participation in ≥ 2 surveillance visits significantly reduced the cumulative CRC incidence (Fig. 3; Table 5).

**Table 5:** Cumulative incidence of colorectal cancer after removal of adenoma with HGD

	*n* (%)	No of person-years	No. of CRCs	Incidence rate per 1000 person-years	5-year cumulative incidence (95% CI)^a^	10-year cumulative incidence (95% CI)^a^	*P* value^b^
Total	813 (100)	2269.9	10	4.4	1.6% (0.7–3.9)	5.5% (2.6–11.4)	
Sex							0.411
Women	306 (37.6)	830.8	5	6.0	2.8% (0.8–9.0)	7.5% (0.3–19.1)	
Men	507 (62.4)	1439.1	5	3.5	1.0% (0.3–3.2)	4.4% (1.4–13.5)	
Age at baseline (years)							0.360
< 55	366 (45.0)	1187.5	5	4.2	1.6% (0.5–5.3)	4.4% (1.6–11.5)	
55–64	242 (29.8)	607.0	2	3.3	0.5% (0.1–3.6)	4.2% (0.8–21.7)	
65–74	155 (19.1)	366.1	2	5.5	2.3% (0.3–15.1)	–	
≥ 75	50 (6.1)	109.4	1	9.1	5.6% (0.8–33.4)	–	
Location of adenomas with HGD^c^							0.267
Only distal	618 (76.0)	1704.7	6	3.5	1.4% (0.4–4.5)	5.6% (2.2–13.5)	
Any proximal	195 (24.0)	565.2	4	7.1	2.5% (0.8–7.9)	5.5% (1.7–17.4)	
Number of adenomas with HGD							0.053
1	729 (89.7)	2014.2	7	3.5	1.3% (0.5–3.9)	4.8% (2.0–11.5)	
≥ 2	84 (10.3)	255.8	3	11.7	4.2% (1.0–16.5)	–	
Size of adenomas with HGD^d^							0.917
< 10 mm	109 (13.4)	327.0	1	3.1	–	6.7% (1.0–38.7)	
10–19 mm	306 (37.6)	871.6	5	5.7	2.1% (0.8–5.9)	–	
≥ 20 mm	398 (49.0)	1071.4	4	3.7	1.7% (0.4–6.7)	5.2% (1.8–14.5)	
Synchronous PMPs^e^							0.057
No	388 (47.7)	1120.9	2	1.8	0.9% (0.1–6.3)	3.1% (0.7–13.3)	
Low-risk polyps	305 (37.5)	790.8	5	6.3	1.4% (0.5–4.3)	6.0% (2.0–16.9)	
High-risk polyps	120 (14.8)	358.2	3	8.4	3.8% (1.0–14.5)	–	
Resection range^f^							0.207
Segmental bowel resection	114 (14.0)	293.9	0	0	–	–	
Local resection	699 (86.0)	1976.1	10	5.1	1.9% (0.8–4.4)	6.5% (3.0–13.4)	
Quality of baseline colonoscopy^g^							0.760
Adequate	745 (91.6)	2093.8	9	4.3	1.5% (0.6–3.8)	5.6% (2.6–11.9)	
Inadequate	68 (8.4)	176.2	1	5.7	3.6% (0.5–22.8)	–	
Baseline serum CEA level							0.014
0–4.9 ng/mL	56 (68.4)	1477.6	4	2.7	0.5% (0.1–2.2)	–	
≥ 5 ng/mL	44 (5.4)	88.9	2	22.5	9.8% (2.2–37.4)	–	
NA	213 (26.2)	703.4	4	5.7	2.5% (0.6–9.8)	9.9% (3.4–27.0)	
Number of surveillance visits							0.002
0	374 (46.0)	557.2	6	10.8	6.4% (2.2–18.0)	–	
1	197 (24.2)	543.2	2	3.7	1.7% (0.2–11.4)	–	
≥ 2	242 (29.8)	1169.6	2	1.7	–	3.0% (0.7–12.2)	

*CRC* colorectal cancer, *CI* confidence interval, *HGD* high-grade dysplasia, *PMP* premalignant polyp, *CEA* carcinoembryonic antigen, *NA* not available

^a^Cumulative incidence was estimated using the Kaplan–Meier method. Some categories of the specified variable could not be estimated

^b^*P* values were calculated with the log-rank test to compare cumulative incidence among each category of the specified variable. Differences with *P* < 0.05 were considered statistically significant

^c^Distal was defined as distal to the splenic flexure. Proximal was defined as proximal to the splenic flexure

^d^Size was defined according to the largest adenomas with HGD seen at baseline

^e^Low-risk polyps were defined as polyps < 10 mm in size and < 5 in number and containing exclusively tubular/hyperplastic histology. High-risk polyps included tubulovillous adenomas, villous adenomas, sessile serrated polyps, traditional serrated adenomas, polyps ≥ 10 mm in size and polyps ≥ 5 in number

^f^Local resection included endoscopic resection (e.g., cold/hot biopsy forceps, endoscopic snare polypectomy, endoscopic mucosal resection, endoscopic submucosal dissection) and transanal local excision

^g^The baseline colonoscopy was inadequate if bowel cleansing was rated “poor” or “very poor”, or if cecal intubation could not be achieved

### Sensitivity analyses

In a sensitivity analysis that included only patients with an adequate baseline colonoscopy, the incidence densities of AN and CRC were 45.2 and 4.3 per 1000 person-years, with a 10-year cumulative incidence of 39.7% and 5.6%, respectively. The risk factors for AN had no change. However, elevated baseline CEA level was no longer a risk factor for CRC (Supplementary Tables S2–S5).

In another sensitivity analysis, we did not exclude patients who developed CRC or adenoma with HGD in the same colonic segment within one year after the baseline colonoscopy. The incidence densities of AN and CRC were 46.1 and 4.8 per 1000 person-years, with a 10-year cumulative incidence of 39.5% and 5.7%, respectively. All risk factors for AN and CRC did not change (Supplementary Tables S6–S9).

## Discussion

Studying the risk of CRC after the removal of adenomas with HGD is a major challenge because this finding is rare at baseline. Many previous studies have confirmed and extended evidence to support that HGD is a risk factor for subsequent AN and CRC [13–17, 26–28], but the incidence of AN or CRC is rarely reported. Therefore, our study provides detailed data on the long-term AN and CRC incidence after the removal of adenomas with HGD by baseline characteristics and surveillance visits. In this study of 814 patients undergoing colonoscopy and adenoma with HGD resection, we found the incidence densities of AN and CRC were 44.3 and 4.4 per 1000 person-years, respectively. Similar incidences have been reported by other high-quality studies, such as Cubiella et al. (AN incidence: 47.1 per 1000 person-years) and Cross et al. (CRC incidence: 3.2 per 1000 person-years) [21, 26]. However, Wieszczy et al. reported a lower CRC incidence (1.5 per 1000 person-years), potentially due to their younger study population compared to ours [28]. We further conducted Kaplan–Meier analysis to estimate the cumulative incidence of AN and CRC after 10 years (39.1% and 5.5%, respectively). The cumulative CRC incidence at 10 years is similar to that reported by Cross et al. (2.7–5.2%) [26], indicating that our findings are reliable. In spite of this, more high-quality studies are warranted to further validate our findings.

Our analyses revealed that synchronous low-risk polyps and high-risk polyps were independent risk factors for AN. In our cohort, approximately 52% of patients had synchronous polyps at baseline. Compared with adenoma with HGD alone, the risk of AN was 1.8-fold higher in patients with synchronous low-risk polyps and 4-fold higher in patients with synchronous high-risk polyps. Previous studies also indicated that multiplicity of polyps at baseline colonoscopy is associated with increased AN risk [18, 19, 21, 29]. Our study provides additional evidence to support this view. It is reasonable to suggest that patients with multiple polyps at baseline may be more susceptible to developing recurrent polyps and subsequent AN [30–33]. Undeniably, the association between synchronous polyps and increased postoperative AN risk may be related to the missed lesions at baseline. A previous study confirmed that multiple polyps at baseline can be associated with a higher probability of missed lesions [34]. However, all colonoscopies in our cohort were performed by experienced endoscopists with high technical proficiency, and the withdrawal time for each colonoscopy exceeded 6 min. And lesions found within 6 months after baseline were classified as baseline lesions rather than newly developed lesions. We believe that the impact of missed lesions at baseline on our results is relatively minor. Additionally, patients with synchronous high-risk polyps had a higher risk of AN compared to those with synchronous low-risk polyps. This can be attributed to the fact that high-risk polyps themselves increase the risk of postoperative AN. Therefore, when both high-risk polyps and adenomas with HGD coexist, the combined effect of these two risk factors further increases AN risk.

We identified that an elevated baseline CEA level was an independent risk factor for CRC, increasing the risk 10-fold compared to normal level. This is a new finding from our study. CEA has long been recognized as a significant prognostic indicator of CRC [35–37], and guidelines recommend monitoring serum CEA levels in CRC patients [38–40]. A previous study showed that colorectal polyps can also cause elevated CEA level [41], but CEA is not yet routinely used as a prognostic indicator for polyp patients. In our study cohort, 73.8% of patients underwent CEA testing at baseline, among whom 5.4% exhibited elevated CEA level. Patients with elevated CEA level are at a significantly higher risk of developing CRC compared to those with normal level. For patients with adenoma with HGD, it is worth considering whether routine CEA testing should be conducted at baseline and whether intensive postoperative surveillance should be implemented for patients with elevated CEA level. Notably, this study revealed that the proportion of dMMR CRC after removal of adenomas with HGD was 73%, a significantly higher percentage than the reported proportion of dMMR in sporadic CRC, which is only 15% [42]. Similarly, Mark et al. discovered that dMMR CRC account for 75% of CRC cases occurring in patients with sessile serrated adenomas with dysplasia [43]. These findings suggest that the occurrence of cytological dysplasia is accompanied by obvious molecular events. Polyps displaying dysplasia are more prone to mismatch repair gene deficiencies, and consequently, more susceptible to developing dMMR CRC.

Current guidelines recommend surveillance colonoscopy within 3 years after removing adenomas with HGD to prevent the occurrence of CRC [10, 11]. Our study showed that surveillance colonoscopy significantly reduced the AN and CRC incidences, with incidences decreasing as surveillance frequency increased. No previous randomized controlled trials directly evaluate the benefits of post-polypectomy surveillance, but some studies have confirmed the need for and effectiveness of surveillance [7, 26]. Cross et al. found patients with adenomas with HGD who did not receive surveillance had a higher CRC incidence than the general population (SIR 1.74, 95% CI 1.21–2.42). In contrast, the CRC incidence was no longer higher (SIR 0.95, 95% CI 0.63–1.38) among patients who underwent at least one surveillance [26]. Similarly, the study by Cottet et al. showed that without surveillance, CRC incidence was 4-fold higher among patients with baseline advanced adenomas than in the general population, but similar with at least one surveillance [7]. Patients with adenoma with HGD have an increased risk of CRC, but this risk is eliminated after at least one surveillance. However, the optimal interval for postoperative surveillance has yet to be determined by either our current study or previous studies. This retrospective design may not be ideal for determining the optimal surveillance interval. Randomized controlled trials, such as EPoS (European Polyp Surveillance) trial and FORTE (5-year or 10-year colonoscopy for 1 to 2 non-advanced adenomas), have been conducted in some European and American countries to provide detailed data on surveillance intervals [44, 45]. Nevertheless, these trials will take several years to complete.

The precise mechanisms underlying the occurrence of metachronous AN and CRC after adenoma removal remain incompletely understood, potentially involving missed baseline lesions, incomplete removal of baseline lesions, and new-onset or sporadic lesions [46]. The optimal preventive strategies for metachronous AN, contingent upon the underlying mechanisms, may vary. If missed baseline lesions are the primary driver, efforts to improve the quality of baseline colonoscopy may be warranted to reduce missed lesions. In cases where incomplete removal is the main cause, focusing on enhancing the technique of polyp removal is crucial. For new-onset or sporadic lesions, improving risk stratification and postoperative surveillance strategies are recommended. The present study focused on AN and CRC that arise from new-onset or sporadic lesions. To minimize the impact of missed lesions, lesions detected within six months following baseline colonoscopy were categorized as baseline lesions rather than new-onset lesions. To reduce the influence of incomplete removal, patients with CRC or adenoma with HGD emerging in the same colon segment within one year after baseline were excluded from the statistical analyses. Thus, the majority of AN and CRC observed in our study can be attributed to new-onset or sporadic lesions. We hope that our findings will provide additional evidence for post-operative surveillance strategies for patients with adenomas with HGD.

The strengths of this study include the utilization of a substantial cohort of meticulously curated data, comprising comprehensive information on baseline and surveillance colonoscopy from 814 patients with adenomas with HGD. In our cohort, more than 90% of baseline colonoscopies were deemed adequate. Furthermore, excluding patients whose baseline colonoscopy was inadequate had minimal impact, suggesting that our findings are relevant in the current era of high-quality colonoscopy.

This study also has some limitations. Firstly, in our study cohort, 392 (48%) patients did not participate in any surveillance, and we did not have information on the reasons for non-participation in surveillance. It was possible that some patients underwent surveillance at hospitals outside of where we obtained data, which could lead to misclassification and an underestimation of the risk associated with surveillance. Secondly, the follow-up rate in our cohort was lower than expected, with 44% of patients excluded due to not undergoing follow-up after baseline, introducing selection bias. However, this follow-up rate was comparable to other studies evaluating the use of surveillance colonoscopy in community settings [21, 47]. Additionally, to mitigate this bias, we conducted two different analyses based on cumulative incidence and incidence density rates. As demonstrated, the results obtained from both analyses showed no significant differences, underscoring the robustness of the study findings. Finally, we have not yet assessed several demographic and epidemiological variables related to the incidence of AN, such as smoking habits, family history of CRC, and body mass index.

In conclusion, patients who had adenoma with HGD removal are at higher risk for AN and CRC. Postoperative surveillance colonoscopy can reduce the risk. Synchronous low-risk polyps and high-risk polyps are risk factors for AN, while elevated baseline serum CEA level is a risk factor for CRC. Patients with these characteristics should receive more frequent surveillance colonoscopy.

### Supplementary Information

Below is the link to the electronic supplementary material.

Supplementary file1 (DOCX 97 kb)
