# Supplementary material for: Risk of advanced neoplasia after removal of colorectal adenomas with high-grade dysplasia
Source: Surg Endosc. 2024 May 28;38(7):3783–98. doi: 10.1007/s00464-024-10898-5 (PMC11219408; doi:10.1007/s00464-024-10898-5)
Supplement: Supplementary file 1 — Supplementary file1 (DOCX 97 kb) [file 464_2024_10898_MOESM1_ESM.docx]

Supplementary Table S1. Follow-up findings and baseline characteristics in the whole cohort.

| **Follow-up findings** | **No colorectal neoplasia**  **(n=550)** | **Non-****advanced neoplasia^a^**  **(n=164)** | **Advanced neoplasia^b^**  **(n=100)** | **Total**  **(n=814)** |
| --- | --- | --- | --- | --- |
| **Follow-up time (person-years)** | 1242.1 | 678.3 | 248.0 | 2168.5 |
| **Median follow-up time (years)** | 1.4(IQR 3.3-0.5) | 3.7(IQR 5.8-2.0) | 1.6(IQR 3.2-0.9) | 1.9(IQR 3.9-0.7) |
| **Median time from baseline to first surveillance (years)** | 0.4(IQR 1.0-0.3) | 0.6(IQR 1.2-0.4) | 0.8(IQR 1.6-0.5) | 0.5(IQR 1.1-0.3) |
| **Sex** |  |  |  |  |
| women | 220(40.0%) | 51(31.1%) | 35(35.0%) | 306（37.6%） |
| men | 330(60.0%) | 113(68.9%) | 65(65.0%) | 508（62.4%） |
| **Age at baseline (years)** |  |  |  |  |
| <55 | 259(47.1%) | 64(39.0%) | 43(43.0%) | 366（45.0%） |
| 55-64 | 161(29.3%) | 56(34.1%) | 25(25.0%) | 242（29.7%） |
| 65-74 | 100(18.2%) | 34(20.7%) | 22(22.0%) | 156（19.2%） |
| ≥75 | 30(5.5%) | 10(6.1%) | 10(10.0%) | 50（6.1%） |
| **Location of adenomas with HGD**^c^ |  |  |  |  |
| Only distal | 431(78.4%) | 116(70.7%) | 72(72.0%) | 619（76.0%） |
| Any proximal | 119(21.6%) | 48(29.3%) | 28(28.0%) | 195（24.0%） |
| **Number of adenomas with HGD** |  |  |  |  |
| 1 | 508(92.4%) | 140(85.4%) | 82(82.0%) | 730（89.7%） |
| ≥2 | 42(7.6%) | 24(14.6%) | 18(18.0%) | 84（10.3%） |
| **Size of adenomas with HGD**^d^ |  |  |  |  |
| <10 mm | 67(12.2%) | 31(18.9%) | 11(11.0%) | 109（13.4%） |
| 10-19 mm | 207(37.6%) | 60(36.6%) | 39(39.0%) | 306（37.6%） |
| ≥20 mm | 276(50.2%) | 73(44.5%) | 50(50.0%) | 399（49.0%） |
| **Synchronous PMPs**^e^ |  |  |  |  |
| No | 292(53.1%) | 66(26.8%) | 31(31.0%) | 389（47.8%） |
| Low-risk polyps | 200(36.4%) | 67(40.9%) | 38(38.0%) | 305（37.5%） |
| High-risk polyps | 58(10.5%) | 31(18.9%) | 31(31.0%) | 120（14.7%） |
| **Resection** **range**^f^ |  |  |  |  |
| Segmental bowel resection | 93(16.9%) | 11(6.7%) | 10(10.0%) | 114（14.0%） |
| Local resection | 457(83.1%) | 153(93.3%) | 90(90.0%) | 700（86.0%） |
| **Quality of baseline colonoscopy**^g^ |  |  |  |  |
| Adequate | 506(92.0%) | 146 (89.0%) | 94(94.0%) | 746（91.6%） |
| Inadequate | 44(8.0%) | 18(11.0%) | 6(6.0%) | 68（8.4%） |
| **Baseline serum CEA level** |  |  |  |  |
| 0-4.9ng/mL | 391(71.1%) | 96(58.5%) | 70(70.0%) | 557（68.4%） |
| ≥5ng/mL | 29(5.3%) | 8(4.9%) | 7(7.0%) | 44（5.4%） |
| NA | 130(23.6%) | 60(36.6%) | 23(23.0%) | 213（26.2%） |
| **Number of surveillance visits** |  |  |  |  |
| 0 | 302(54.9%) | 40(24.4%) | 53(53.0%) | 395（48.5%） |
| 1 | 121(22.0%) | 44(26.8%) | 34(34.0%) | 199（24.4%） |
| ≥2 | 127(23.1%) | 80(48.8%) | 13(13.0%) | 220（27.0%） |

^a^Non-advanced neoplasia was defined as tubular adenoma <10mm without HGD, and serrated polyps <10mm without any grade of dysplasia.

^b^Advanced neoplasia was defined as CRC, advanced adenomas (≥10mm, or containing tubulovillous/villous histology or HGD), and advanced serrated polyps (≥10mm or containing any grade of dysplasia).

^c^Distal was defined as distal to the splenic flexure. Proximal was defined as proximal to the splenic flexure.

^d^Size was defined according to the largest adenomas with HGD seen at baseline.

^e^Low-risk polyps were defined as polyps <10mm in size and <5 in number and containing exclusively tubular/hyperplastic histology. High-risk polyps included tubulovillous adenomas, villous adenomas, sessile serrated polyps, traditional serrated adenomas, polyps ≥10mm in size and polyps ≥5 in number.

^f^Local resection included endoscopic resection (eg, cold/hot biopsy forceps, endoscopic snare polypectomy, endoscopic mucosal resection, endoscopic submucosal dissection) and transanal local excision.

^g^The baseline colonoscopy was inadequate if bowel cleansing was rated "poor" or "very poor", or if cecal intubation could not be achieved.

Abbreviations: IQR, interquartile range; HGD, high-grade dysplasia; PMP, premalignant polyp; CEA, carcinoembryonic antigen; NA, not available.

Supplementary Table S2. Long-term incidence of advanced neoplasia by baseline characteristics and number of surveillance visits, including only patients with an adequate baseline colonoscopy.

|  | **n (%)** | **No of person-years** | **No of ANs** | **Incidence rate per 1000 person-years** | **Univariable HR (95% CI)** | ***P* value^a^** | **Multivariable HR (95% CI)** | ***P* value^a^** |
| --- | --- | --- | --- | --- | --- | --- | --- | --- |
| **Total** | 742(100%) | 1992.7 | 90 | 45.2 |  |  |  |  |
| **Sex** |  |  |  |  |  |  |  |  |
| women | 283(38.1%) | 738.6 | 32 | 43.3 | 1(ref) |  |  |  |
| men | 459(61.9%) | 1254.1 | 58 | 46.2 | 1.06(0.69-1.64) | 0.781 |  |  |
| **Age at baseline (years)** |  |  |  |  |  |  |  |  |
| <55 | 345(46.5%) | 1061.9 | 41 | 38.6 | 1(ref) |  | 1(ref) |  |
| 55-64 | 221(29.8%) | 534.9 | 22 | 41.1 | 1.08(0.64-1.81) | 0.777 | 0.93(0.54-1.58) | 0.773 |
| 65-74 | 136(18.3%) | 305.5 | 18 | 58.9 | 1.57(0.89-2.74) | 0.117 | 1.19(0.67-2.12) | 0.560 |
| ≥75 | 40(5.4%) | 90.5 | 9 | 99.4 | 2.64(1.28-5.46) | 0.009 | 1.48(0.70-3.11) | 0.305 |
| **Location of adenomas with HGD**^b^ |  |  |  |  |  |  |  |  |
| Only distal | 562(75.7%) | 1498.4 | 64 | 42.7 | 1(ref) |  |  |  |
| Any proximal | 180(24.3%) | 494.3 | 26 | 52.6 | 1.23(0.78-1.94) | 0.377 |  |  |
| **Number of adenomas with HGD** |  |  |  |  |  |  |  |  |
| 1 | 668(90.0%) | 1778.2 | 74 | 41.6 | 1(ref) |  | 1(ref) |  |
| ≥2 | 74(10.0%) | 214.6 | 16 | 74.6 | 1.76(1.02-3.02) | 0.041 | 1.49(0.86-2.58) | 0.152 |
| **Size of adenomas with HGD**^c^ |  |  |  |  |  |  |  |  |
| <10 mm | 101(13.6%) | 297.3 | 10 | 33.6 | 1(ref) |  |  |  |
| 10-19 mm | 284(38.3%) | 780.3 | 36 | 46.1 | 1.37(0.68-2.76) | 0.383 |  |  |
| ≥20 mm | 357(48.1%) | 915.2 | 44 | 48.1 | 1.46(0.73-2.90) | 0.281 |  |  |
| **Synchronous PMPs**^d^ |  |  |  |  |  |  |  |  |
| No | 366(49.3%) | 991.8 | 29 | 29.2 | 1(ref) |  | 1(ref) |  |
| Low-risk polyps | 272(36.7%) | 707.7 | 36 | 50.9 | 1.74(1.07-2.83) | 0.027 | 1.80(1.10-2.95) | 0.019 |
| High-risk polyps | 104(14.0%) | 293.2 | 25 | 85.3 | 2.88(1.69-4.92) | ＜0.001 | 3.42(1.99-5.89) | ＜0.001 |
| **Resection** **range**^e^ |  |  |  |  |  |  |  |  |
| Segmental bowel resection | 99(13.3%) | 251.6 | 9 | 35.8 | 1(ref) |  |  |  |
| Local resection | 643(86.7%) | 1741.1 | 81 | 46.5 | 1.29(0.65-2.58) | 0.468 |  |  |
| **Baseline serum CEA level** |  |  |  |  |  |  |  |  |
| 0-4.9ng/mL | 510(68.7%) | 1296.4 | 64 | 49.4 | 1(ref) |  |  |  |
| ≥5ng/mL | 36(4.9%) | 67.7 | 6 | 88.6 | 1.83(0.79-4.23) | 0.158 |  |  |
| NA | 196(26.4%) | 628.7 | 20 | 31.8 | 0.64(0.39-1.05) | 0.078 |  |  |
| **Number of surveillance visits** |  |  |  |  |  |  |  |  |
| 0 | 357(48.1%) | 519.9 | 47 | 90.4 | 1(ref) |  | 1(ref) |  |
| 1 | 181(24.4%) | 481.0 | 32 | 66.5 | 0.66(0.42-1.04) | 0.075 | 0.60(0.38-0.94) | 0.027 |
| ≥2 | 204(27.5%) | 991.8 | 11 | 11.1 | 0.10(0.05-0.20) | ＜0.001 | 0.09(0.05-0.18) | ＜0.001 |

^a^*P* values were calculated with the likelihood ratio test. Differences with P<0.05 were considered statistically significant.

^b^Distal was defined as distal to the splenic flexure. Proximal was defined as proximal to the splenic flexure.

^c^Size was defined according to the largest adenomas with HGD seen at baseline.

^d^Low-risk polyps were defined as polyps <10mm in size and <5 in number and containing exclusively tubular/hyperplastic histology. High-risk polyps included tubulovillous adenomas, villous adenomas, sessile serrated polyps, traditional serrated adenomas, polyps ≥10mm in size and polyps ≥5 in number.

^e^Local resection included endoscopic resection (eg, cold/hot biopsy forceps, endoscopic snare polypectomy, endoscopic mucosal resection, endoscopic submucosal dissection) and transanal local excision.

Abbreviations: AN, advanced neoplasia; HR, hazard ratio; CI, confidence interval; HGD, high-grade dysplasia; PMP, premalignant polyp; CEA, carcinoembryonic antigen; NA, not available.

Supplementary Table S3. Cumulative incidence of advanced neoplasia, including only patients with an adequate baseline colonoscopy.

|  | **n (%)** | **No of**  **person-years** | **No of ANs** | **Incidence rate per 1000 person-years** | **5-year cumulative incidence (95% CI)^a^** | **10-year cumulative incidence (95% CI)^a^** | ***P* value^b^** |
| --- | --- | --- | --- | --- | --- | --- | --- |
| **Total** | 742(100%) | 1992.7 | 90 | 45.2 | 20.3% (16.3-25.2) | 39.7% (30.0-54.2) |  |
| **Sex** |  |  |  |  |  |  | 0.781 |
| women | 283(38.1%) | 738.6 | 32 | 43.3 | 20.9% (14.4-29.7) | 33.7% (21.8-49.7) |  |
| men | 459(61.9%) | 1254.1 | 58 | 46.2 | 20.0% (15.1-26.1) | 44.0% (27.1-65.5) |  |
| **Age at baseline (years)** |  |  |  |  |  |  | 0.010 |
| <55 | 345(46.5%) | 1061.9 | 41 | 38.6 | 16.8% (11.9-23.3) | 35.4% (21.1-55.2) |  |
| 55-64 | 221(29.8%) | 534.9 | 22 | 41.1 | 18.7% (11.8-28.9) | 35.1% (19.6-57.5) |  |
| 65-74 | 136(18.3%) | 305.5 | 18 | 58.9 | 24.3% (14.9-38.0) | － |  |
| ≥75 | 40(5.4%) | 90.5 | 9 | 99.4 | 48.9% (27.3-75.7) | － |  |
| **Location of adenomas with HGD**^c^ |  |  |  |  |  |  | 0.376 |
| Only distal | 562(75.7%) | 1498.4 | 64 | 42.7 | 19.4% (14.8-25.0) | 43.4% (28.0-62.7) |  |
| Any proximal | 180(24.3%) | 494.3 | 26 | 52.6 | 22.9% (15.2-33.6) | 31.0% (19.4-47.1) |  |
| **Number of adenomas with HGD** |  |  |  |  |  |  | 0.038 |
| 1 | 668(90.0%) | 1778.2 | 74 | 41.6 | 18.4% (14.4-23.3) | 35.8% (23.5-51.9) |  |
| ≥2 | 74(10.0%) | 214.6 | 16 | 74.6 | 33.5% (20.0-52.5) | － |  |
| **Size of adenomas with HGD**^d^ |  |  |  |  |  |  | 0.323 |
| <10 mm | 101(13.6%) | 297.3 | 10 | 33.6 | 13.5% (6.8-25.7) | 27.0% (12.9-51.1) |  |
| 10-19 mm | 284(38.3%) | 780.3 | 36 | 46.1 | 19.7% (13.6-28.1) | － |  |
| ≥20 mm | 357(48.1%) | 915.2 | 44 | 48.1 | 23.1% (17.0-30.9) | 44.3% (25.4-68.9) |  |
| **Synchronous PMPs**^e^ |  |  |  |  |  |  | ＜0.001 |
| No | 366(49.3%) | 991.8 | 29 | 29.2 | 12.8% (8.4-19.2) | 36.9% (17.8-66.1) |  |
| Low-risk polyps | 272(36.7%) | 707.7 | 36 | 50.9 | 21.2% (15.0-29.5) | 37.6% (22.7-57.9) |  |
| High-risk polyps | 104(14.0%) | 293.2 | 25 | 85.3 | 39.0% (27.0-54.1) | － |  |
| **Resection** **range**^f^ |  |  |  |  |  |  | 0.467 |
| Segmental bowel resection | 99(13.3%) | 251.6 | 9 | 35.8 | 13.3% (6.3-26.6) | － |  |
| Local resection | 643(86.7%) | 1741.1 | 81 | 46.5 | 21.2% (16.8-26.6) | 36.9% (27.6-48.1) |  |
| **Baseline serum CEA level** |  |  |  |  |  |  | 0.047 |
| 0-4.9ng/mL | 510(68.7%) | 1296.4 | 64 | 49.4 | 21.1% (16.1-27.2) | － |  |
| ≥5ng/mL | 36(4.9%) | 67.7 | 6 | 88.6 | 24.3% (10.5-50.3) | － |  |
| NA | 196(26.4%) | 628.7 | 20 | 31.8 | 17.4% (11.0-26.9) | 24.3% (14.8-38.4) |  |
| **Number of surveillance visits** |  |  |  |  |  |  | ＜0.001 |
| 0 | 357(48.1%) | 519.9 | 47 | 90.4 | 35.6% (26.4-46.9) | － |  |
| 1 | 181(24.4%) | 481.0 | 32 | 66.5 | 30.3% (20.6-43.2) | － |  |
| ≥2 | 204(27.5%) | 991.8 | 11 | 11.1 | 6.0% (3.0-12.0) | 13.3% (6.4-26.7) |  |

^a^Cumulative incidence was estimated using the Kaplan-Meier method. Some categories of the specified variable could not be estimated.

^b^*P* values were calculated with the log-rank test to compare cumulative incidence among each category of the specified variable. Differences with P<0.05 were considered statistically significant.

^c^Distal was defined as distal to the splenic flexure. Proximal was defined as proximal to the splenic flexure.

^d^Size was defined according to the largest adenomas with HGD seen at baseline.

^e^Low-risk polyps were defined as polyps <10mm in size and <5 in number and containing exclusively tubular/hyperplastic histology. High-risk polyps included tubulovillous adenomas, villous adenomas, sessile serrated polyps, traditional serrated adenomas, polyps ≥10mm in size and polyps ≥5 in number.

^f^Local resection included endoscopic resection (eg, cold/hot biopsy forceps, endoscopic snare polypectomy, endoscopic mucosal resection, endoscopic submucosal dissection) and transanal local excision.

Abbreviations: AN, advanced neoplasia; CI, confidence interval; HGD, high-grade dysplasia; PMP, premalignant polyp; CEA, carcinoembryonic antigen; NA, not available.

Supplementary Table S4. Long-term incidence of colorectal cancer by baseline characteristics and number of surveillance visits, including only patients with an adequate baseline colonoscopy.

|  | **n (%)** | **No of person-years** | **No of CRCs** | **Incidence rate per 1000 person-years** | **Univariable HR (95% CI)** | ***P* value^a^** |
| --- | --- | --- | --- | --- | --- | --- |
| **Total** | 745(100%) | 2093.8 | 9 | 4.3 |  |  |
| **Sex** |  |  |  |  |  |  |
| women | 286(38.4%) | 771.1 | 5 | 6.5 | 1(ref) |  |
| men | 459(61.6%) | 1322.7 | 4 | 3.0 | 0.48(0.13-1.80) | 0.278 |
| **Age at baseline (years)** |  |  |  |  |  |  |
| <55 | 347(46.6%) | 1117.2 | 5 | 4.5 | 1(ref) |  |
| 55-64 | 221(29.7%) | 552.0 | 2 | 3.6 | 0.98(0.19-5.12) | 0.984 |
| 65-74 | 137(18.4%) | 329.8 | 2 | 6.1 | 1.68(0.32-8.80) | 0.539 |
| ≥75 | 40(5.4%) | 94.8 | 0 | 0 | － | 0.987 |
| **Location of adenomas with HGD**^b^ |  |  |  |  |  |  |
| Only distal | 565(75.8%) | 1568.1 | 6 | 3.8 | 1(ref) |  |
| Any proximal | 180(24.2%) | 525.7 | 3 | 5.7 | 1.48(0.37-5.92) | 0.579 |
| **Number of adenomas with HGD** |  |  |  |  |  |  |
| 1 | 671(90.1%) | 1863.8 | 7 | 3.8 | 1(ref) |  |
| ≥2 | 74(9.9%) | 230.0 | 2 | 8.7 | 2.38(0.49-11.46) | 0.281 |
| **Size of adenomas with HGD**^c^ |  |  |  |  |  |  |
| <10 mm | 101(13.6%) | 311.8 | 1 | 3.2 | 1(ref) |  |
| 10-19 mm | 286(38.4%) | 818.2 | 5 | 6.1 | 2.19(0.25-18.86) | 0.477 |
| ≥20 mm | 358(48.1%) | 963.8 | 3 | 3.1 | 1.04(0.11-10.00) | 0.975 |
| **Synchronous PMPs**^d^ |  |  |  |  |  |  |
| No | 367(49.3%) | 1045.6 | 2 | 1.9 | 1(ref) |  |
| Low-risk polyps | 273(36.6%) | 733.3 | 5 | 6.8 | 3.83(0.74-19.78) | 0.109 |
| High-risk polyps | 105(14.1%) | 314.9 | 2 | 6.4 | 3.37(0.48-23.97) | 0.224 |
| **Resection** **range**^e^ |  |  |  |  |  |  |
| Segmental bowel resection | 99(13.3%) | 266.0 | 0 | 0 | 1(ref) |  |
| Local resection | 646(86.7%) | 1827.8 | 9 | 4.9 | 25.49 (0.01-93958.28) | 0.440 |
| **Baseline serum CEA level** |  |  |  |  |  |  |
| 0-4.9ng/mL | 512(68.7%) | 1362.4 | 4 | 2.9 | 1(ref) |  |
| ≥5ng/mL | 36 (4.8%) | 81.2 | 1 | 12.3 | 4.47(0.50-40.10) | 0.181 |
| NA | 197(26.4%) | 650.2 | 4 | 6.2 | 2.04(0.51-8.16) | 0.314 |
| **Number of surveillance visits** |  |  |  |  |  |  |
| 0 | 340(45.6%) | 499.9 | 5 | 10.0 | 1(ref) |  |
| 1 | 179(24.0%) | 492.3 | 2 | 4.1 | 0.38(0.07-1.97) | 0.249 |
| ≥2 | 226(30.3%) | 1101.6 | 2 | 1.8 | 0.13(0.02-0.67) | 0.015 |

^a^*P* values were calculated with the likelihood ratio test. Differences with P<0.05 were considered statistically significant.

^b^Distal was defined as distal to the splenic flexure. Proximal was defined as proximal to the splenic flexure.

^c^Size was defined according to the largest adenomas with HGD seen at baseline.

^d^Low-risk polyps were defined as polyps <10mm in size and <5 in number and containing exclusively tubular/hyperplastic histology. High-risk polyps included tubulovillous adenomas, villous adenomas, sessile serrated polyps, traditional serrated adenomas, polyps ≥10mm in size and polyps ≥5 in number.

^e^Local resection included endoscopic resection (eg, cold/hot biopsy forceps, endoscopic snare polypectomy, endoscopic mucosal resection, endoscopic submucosal dissection) and transanal local excision.

Abbreviations: CRC, colorectal cancer; HR, hazard ratio; CI, confidence interval; HGD, high-grade dysplasia; PMP, premalignant polyp; CEA, carcinoembryonic antigen; NA, not available.

Supplementary Table S5. Cumulative incidence of colorectal cancer, including only patients with an adequate baseline colonoscopy.

|  | **n (%)** | **No of**  **person-years** | **No of CRCs** | **Incidence rate per 1000 person-years** | **5-year cumulative incidence (95% CI)^a^** | **10-year cumulative incidence (95% CI)^a^** | ***P* value^b^** |
| --- | --- | --- | --- | --- | --- | --- | --- |
| **Total** | 745(100%) | 2093.8 | 9 | 4.3 | 1.5% (0.5-3.8) | 5.6% (2.6-11.9) |  |
| **Sex** |  |  |  |  |  |  | 0.267 |
| women | 286(38.4%) | 771.1 | 5 | 6.5 | 3.0% (0.9-9.9) | 8.2% (3.1-20.8) |  |
| men | 459(61.6%) | 1322.7 | 4 | 3.0 | 0.6% (0.1-2.3) | 4.2% (1.2-14.0) |  |
| **Age at baseline (years)** |  |  |  |  |  |  | 0.874 |
| <55 | 347(46.6%) | 1117.2 | 5 | 4.5 | 1.7% (0.5-5.6) | 4.6% (1.7-12.1) |  |
| 55-64 | 221(29.7%) | 552.0 | 2 | 3.6 | 0.6% (0.1-3.9) | 4.7% (0.8-24.1) |  |
| 65-74 | 137(18.4%) | 329.8 | 2 | 6.1 | 2.5% (0.4-16.5) | － |  |
| ≥75 | 40(5.4%) | 94.8 | 0 | 0 | － | － |  |
| **Location of adenomas with HGD**^c^ |  |  |  |  |  |  | 0.577 |
| Only distal | 565(75.8%) | 1568.1 | 6 | 3.8 | 1.5% (0.5-4.9) | 6.0% (2.4-14.4) |  |
| Any proximal | 180(24.2%) | 525.7 | 3 | 5.7 | 1.4% (0.4-5.4) | 4.6% (1.1-17.9) |  |
| **Number of adenomas with HGD** |  |  |  |  |  |  | 0.266 |
| 1 | 671(90.1%) | 1863.8 | 7 | 3.8 | 1.5% (0.5-4.2) | 5.1% (2.1-12.1) |  |
| ≥2 | 74(9.9%) | 230.0 | 2 | 8.7 | 1.7% (0.2-11.4) | － |  |
| **Size of adenomas with HGD**^d^ |  |  |  |  |  |  | 0.730 |
| <10 mm | 101(13.6%) | 311.8 | 1 | 3.2 | － | 6.7% (1.0-38.7) |  |
| 10-19 mm | 286(38.4%) | 818.2 | 5 | 6.1 | 2.2% (0.8-6.3) | － |  |
| ≥20 mm | 358(48.1%) | 963.8 | 3 | 3.1 | 1.2% (0.2-7.9) | 5.2% (1.6-16.2) |  |
| **Synchronous PMPs**^e^ |  |  |  |  |  |  | 0.139 |
| No | 367(49.3%) | 1045.6 | 2 | 1.9 | 1.0% (0.1-7.0) | 3.4% (0.8-14.2) |  |
| Low-risk polyps | 273(36.6%) | 733.3 | 5 | 6.8 | 1.5% (0.5-4.6) | 6.3% (2.2-17.7) |  |
| High-risk polyps | 105(14.1%) | 314.9 | 2 | 6.4 | 2.4% (0.4-16.1) | － |  |
| **Resection** **range**^f^ |  |  |  |  |  |  | 0.225 |
| Segmental bowel resection | 99(13.3%) | 266.0 | 0 | 0 | － | － |  |
| Local resection | 646(86.7%) | 1827.8 | 9 | 4.9 | 1.8% (0.6-4.4) | 6.6% (3.0-14.0) |  |
| **Baseline serum CEA level** |  |  |  |  |  |  | 0.297 |
| 0-4.9ng/mL | 512(68.7%) | 1362.4 | 4 | 2.9 | 0.6% (0.2-2.4) | － |  |
| ≥5ng/mL | 36 (4.8%) | 81.2 | 1 | 12.3 | 3.5% (0.5-22.1) | － |  |
| NA | 197(26.4%) | 650.2 | 4 | 6.2 | 2.8% (0.7-10.8) | 10.6% (3.7-28.5) |  |
| **Number of surveillance visits** |  |  |  |  |  |  | 0.006 |
| 0 | 340(45.6%) | 499.9 | 5 | 10.0 | 5.5% (1.5-19.2) | － |  |
| 1 | 179(24.0%) | 492.3 | 2 | 4.1 | 1.9% (0.3-12.7) | － |  |
| ≥2 | 226(30.3%) | 1101.6 | 2 | 1.8 | － | 3.1% (0.8-12.4) |  |

^a^Cumulative incidence was estimated using the Kaplan-Meier method. Some categories of the specified variable could not be estimated.

^b^*P* values were calculated with the log-rank test to compare cumulative incidence among each category of the specified variable. Differences with P<0.05 were considered statistically significant.

^c^Distal was defined as distal to the splenic flexure. Proximal was defined as proximal to the splenic flexure.

^d^Size was defined according to the largest adenomas with HGD seen at baseline.

^e^Low-risk polyps were defined as polyps <10mm in size and <5 in number and containing exclusively tubular/hyperplastic histology. High-risk polyps included tubulovillous adenomas, villous adenomas, sessile serrated polyps, traditional serrated adenomas, polyps ≥10mm in size and polyps ≥5 in number.

^f^Local resection included endoscopic resection (eg, cold/hot biopsy forceps, endoscopic snare polypectomy, endoscopic mucosal resection, endoscopic submucosal dissection) and transanal local excision.

Abbreviations: CRC, colorectal cancer; CI, confidence interval; HGD, high-grade dysplasia; PMP, premalignant polyp; CEA, carcinoembryonic antigen; NA, not available.

Supplementary Table S6. Long-term incidence of advanced neoplasia by baseline characteristics and number of surveillance visits, without excluding patients who developed colorectal cancers or adenomas with high-grade dysplasia in the same colonic segment within one year after the baseline colonoscopy.

|  | **n (%)** | **No of person-years** | **No of ANs** | **Incidence rate per 1000 person-years** | **Univariable HR (95% CI)** | ***P* value^a^** | **Multivariable HR (95% CI)** | ***P* value^a^** |
| --- | --- | --- | --- | --- | --- | --- | --- | --- |
| **Total** | 814(100%) | 2168.5 | 100 | 46.1 |  |  |  |  |
| **Sex** |  |  |  |  |  |  |  |  |
| women | 306(37.6%) | 800.1 | 35 | 43.7 | 1(ref) |  |  |  |
| men | 508(62.4%) | 1368.4 | 65 | 47.5 | 1.08(0.72-1.63) | 0.709 |  |  |
| **Age at baseline (years)** |  |  |  |  |  |  |  |  |
| <55 | 366(45.0%) | 1133.3 | 43 | 37.9 | 1(ref) |  | 1(ref) |  |
| 55-64 | 242(29.7%) | 586.9 | 25 | 42.6 | 1.14(0.69-1.86) | 0.615 | 0.99(0.59-1.62) | 0.958 |
| 65-74 | 156(19.2%) | 343.2 | 22 | 64.1 | 1.73(1.03-2.91) | 0.038 | 1.32(0.78-2.26) | 0.303 |
| ≥75 | 50(6.1%) | 105.1 | 10 | 95.1 | 2.56(1.28-5.13) | 0.008 | 1.37(0.67-2.82) | 0.390 |
| **Location of adenomas with HGD**^b^ |  |  |  |  |  |  |  |  |
| Only distal | 619(76.0%) | 1636.3 | 72 | 44.0 | 1(ref) |  |  |  |
| Any proximal | 195(24.0%) | 532.2 | 28 | 52.6 | 1.19(0.77-1.85) | 0.429 |  |  |
| **Number of adenomas with HGD** |  |  |  |  |  |  |  |  |
| 1 | 730(89.7%) | 1928.1 | 82 | 42.5 | 1(ref) |  | 1(ref) |  |
| ≥2 | 84(10.3%) | 240.4 | 18 | 74.9 | 1.74(1.04-2.90) | 0.034 | 1.48(0.88-2.48) | 0.137 |
| **Size of adenomas with HGD**^c^ |  |  |  |  |  |  |  |  |
| <10 mm | 109(13.4%) | 312.4 | 11 | 35.2 | 1(ref) |  |  |  |
| 10-19 mm | 306(37.6%) | 833.1 | 39 | 46.8 | 1.32(0.67-2.58) | 0.418 |  |  |
| ≥20 mm | 399(49.0%) | 1022.9 | 50 | 48.9 | 1.41(0.73-2.71) | 0.302 |  |  |
| **Synchronous PMPs**^d^ |  |  |  |  |  |  |  |  |
| No | 389(47.8%) | 1068.6 | 31 | 29.0 | 1(ref) |  | 1(ref) |  |
| Low-risk polyps | 305(37.5%) | 764.4 | 38 | 49.7 | 1.71(1.06-2.75) | 0.027 | 1.72(1.07-2.77) | 0.025 |
| High-risk polyps | 120(14.7%) | 335.4 | 31 | 92.4 | 3.15(1.92-5.19) | ＜0.001 | 3.86(2.32-6.40) | 0.026 |
| **Resection** **range**^e^ |  |  |  |  |  |  |  |  |
| Segmental bowel resection | 114(14.0%) | 278.2 | 10 | 35.9 | 1(ref) |  |  |  |
| Local resection | 700(86.0%) | 1890.2 | 90 | 47.6 | 1.31(0.68-2.25) | 0.419 |  |  |
| **Quality of baseline colonoscopy**^f^ |  |  |  |  |  |  |  |  |
| Adequate | 746(91.6%) | 1995.3 | 94 | 47.1 | 1(ref) |  |  |  |
| Inadequate | 68(8.4%) | 173.2 | 6 | 34.6 | 0.76(0.33-1.72) | 0.504 |  |  |
| **Baseline serum CEA level** |  |  |  |  |  |  |  |  |
| 0-4.9ng/mL | 557(68.4%) | 1410.6 | 70 | 49.6 | 1(ref) |  |  |  |
| ≥5ng/mL | 44(5.4%) | 75.4 | 7 | 92.8 | 1.90(0.87-4.15) | 0.106 |  |  |
| NA | 213(26.2%) | 682.5 | 23 | 33.7 | 0.67(0.42-1.08) | 0.100 |  |  |
| **Number of surveillance visits** |  |  |  |  |  |  |  |  |
| 0 | 395(48.5%) | 579.8 | 53 | 91.4 | 1(ref) |  | 1(ref) |  |
| 1 | 199(24.4%) | 530.2 | 34 | 64.1 | 0.64(0.41-0.99) | 0.044 | 0.55(0.36-0.86) | 0.009 |
| ≥2 | 220(27.0%) | 1058.5 | 13 | 12.3 | 0.12(0.06-0.21) | ＜0.001 | 0.10(0.05-0.19) | ＜0.001 |

^a^*P* values were calculated with the likelihood ratio test. Differences with P<0.05 were considered statistically significant.

^b^Distal was defined as distal to the splenic flexure. Proximal was defined as proximal to the splenic flexure.

^c^Size was defined according to the largest adenomas with HGD seen at baseline.

^d^Low-risk polyps were defined as polyps <10mm in size and <5 in number and containing exclusively tubular/hyperplastic histology. High-risk polyps included tubulovillous adenomas, villous adenomas, sessile serrated polyps, traditional serrated adenomas, polyps ≥10mm in size and polyps ≥5 in number.

^e^Local resection included endoscopic resection (eg, cold/hot biopsy forceps, endoscopic snare polypectomy, endoscopic mucosal resection, endoscopic submucosal dissection) and transanal local excision.

^f^The baseline colonoscopy was inadequate if bowel cleansing was rated "poor" or "very poor", or if cecal intubation could not be achieved.

Abbreviations: AN, advanced neoplasia; HR, hazard ratio; CI, confidence interval; HGD, high-grade dysplasia; PMP, premalignant polyp; CEA, carcinoembryonic antigen; NA, not available.

Supplementary Table S7. Cumulative incidence of advanced neoplasia, without excluding patients who developed colorectal cancers or adenomas with high-grade dysplasia in the same colonic segment within one year after the baseline colonoscopy.

|  | **n (%)** | **No of**  **person-years** | **No of ANs** | **Incidence rate per 1000 person-years** | **5-year cumulative incidence (95% CI)^a^** | **10-year cumulative incidence (95% CI)^a^** | ***P* value^b^** |
| --- | --- | --- | --- | --- | --- | --- | --- |
| **Total** | 814(100%) | 2168.5 | 100 | 46.1 | 20.3% (16.4-24.8) | 39.5% (28.4-53.1) |  |
| **Sex** |  |  |  |  |  |  | 0.709 |
| women | 306(37.6%) | 800.1 | 35 | 43.7 | 20.4% (14.3-28.6) | 32.4% (21.1-47.7) |  |
| men | 508(62.4%) | 1368.4 | 65 | 47.5 | 20.2% (15.6-26.0) | 45.1% (28.3-66.0) |  |
| **Age at baseline (years)** |  |  |  |  |  |  | 0.003 |
| <55 | 366(45.0%) | 1133.3 | 43 | 37.9 | 16.3% (117-22.5) | 33.7% (20.4-52.3) |  |
| 55-64 | 242(29.7%) | 586.9 | 25 | 42.6 | 18.2% (11.8-27.5) | 38.0% (22.1-59.9) |  |
| 65-74 | 156(19.2%) | 343.2 | 22 | 64.1 | 26.0% (16.9-38.8) | － |  |
| ≥75 | 50(6.1%) | 105.1 | 10 | 95.1 | 46.7% (26.9-71.7) | － |  |
| **Location of adenomas with HGD**^c^ |  |  |  |  |  |  | 0.428 |
| Only distal | 619(76.0%) | 1636.3 | 72 | 44.0 | 19.4% (15.1-24.7) | 42.8% (28.6-60.5) |  |
| Any proximal | 195(24.0%) | 532.2 | 28 | 52.6 | 22.8% (15.5-32.9) | 30.8% (19.5-46.6) |  |
| **Number of adenomas with HGD** |  |  |  |  |  |  | 0.032 |
| 1 | 730(89.7%) | 1928.1 | 82 | 42.5 | 18.5% (14.7-23.1) | 34.8% (23.3-49.9) |  |
| ≥2 | 84(10.3%) | 240.4 | 18 | 74.9 | 32.1% (19.6-49.6) | － |  |
| **Size of adenomas with HGD**^d^ |  |  |  |  |  |  | 0.338 |
| <10 mm | 109(13.4%) | 312.4 | 11 | 35.2 | 14.9% (7.9-27.2) | 28.2% (14.0-51.7) |  |
| 10-19 mm | 306(37.6%) | 833.1 | 39 | 46.8 | 19.4% (13.6-27.2) | － |  |
| ≥20 mm | 399(49.0%) | 1022.9 | 50 | 48.9 | 22.6% (17.0-29.8) | 42.8% (25.9-64.7) |  |
| **Synchronous PMPs**^e^ |  |  |  |  |  |  | ＜0.001 |
| No | 389(47.8%) | 1068.6 | 31 | 29.0 | 12.4% (8.3-18.4) | 36.2% (17.2-65.7) |  |
| Low-risk polyps | 305(37.5%) | 764.4 | 38 | 49.7 | 20.7% (14.7-28.6) | 36.0% (22.1-55.1) |  |
| High-risk polyps | 120(14.7%) | 335.4 | 31 | 92.4 | 39.3% (28.2-52.9) | － |  |
| **Resection** **range**^f^ |  |  |  |  |  |  | 0.418 |
| Segmental bowel resection | 114(14.0%) | 278.2 | 10 | 35.9 | 13.7% (6.9-26.2) | － |  |
| Local resection | 700(86.0%) | 1890.2 | 90 | 47.6 | 21.2% (17.0-26.2) | 37.0% (28.0-47.7) |  |
| **Quality of baseline colonoscopy**^g^ |  |  |  |  |  |  | 0.503 |
| Adequate | 746(91.6%) | 1995.3 | 94 | 47.1 | 20.8% (16.8-25.7) | 40.1% (28.4-54.5) |  |
| Inadequate | 68(8.4%) | 173.2 | 6 | 34.6 | 13.9% (5.9-31.0) | － |  |
| **Baseline serum CEA level** |  |  |  |  |  |  | 0.039 |
| 0-4.9ng/mL | 557(68.4%) | 1410.6 | 70 | 49.6 | 21.0% (16.3-26.8) | － |  |
| ≥5ng/mL | 44(5.4%) | 75.4 | 7 | 92.8 | 30.1% (13.9-57.6) | － |  |
| NA | 213(26.2%) | 682.5 | 23 | 33.7 | 16.9% (10.9-25.7) | 26.4% (16.5-40.7) |  |
| **Number of surveillance visits** |  |  |  |  |  |  | ＜0.001 |
| 0 | 395(48.5%) | 579.8 | 53 | 91.4 | 35.2% (26.7-45.5) | － |  |
| 1 | 199(24.4%) | 530.2 | 34 | 64.1 | 29.2% (20.2-41.1) | － |  |
| ≥2 | 220(27.0%) | 1058.5 | 13 | 12.3 | 6.0% (3.1-11.6) | 15.0% (7.6-28.3) |  |

^a^Cumulative incidence was estimated using the Kaplan-Meier method. Some categories of the specified variable could not be estimated.

^b^*P* values were calculated with the log-rank test to compare cumulative incidence among each category of the specified variable. Differences with P<0.05 were considered statistically significant.

^c^Distal was defined as distal to the splenic flexure. Proximal was defined as proximal to the splenic flexure.

^d^Size was defined according to the largest adenomas with HGD seen at baseline.

^e^Low-risk polyps were defined as polyps <10mm in size and <5 in number and containing exclusively tubular/hyperplastic histology. High-risk polyps included tubulovillous adenomas, villous adenomas, sessile serrated polyps, traditional serrated adenomas, polyps ≥10mm in size and polyps ≥5 in number.

^f^Local resection included endoscopic resection (eg, cold/hot biopsy forceps, endoscopic snare polypectomy, endoscopic mucosal resection, endoscopic submucosal dissection) and transanal local excision.

^g^The baseline colonoscopy was inadequate if bowel cleansing was rated "poor" or "very poor", or if cecal intubation could not be achieved.

Abbreviations: AN, advanced neoplasia; CI, confidence interval; HGD, high-grade dysplasia; PMP, premalignant polyp; CEA, carcinoembryonic antigen; NA, not available.

Supplementary Table S8. Long-term incidence of colorectal cancer by baseline characteristics and number of surveillance visits, without excluding patients who developed colorectal cancers or adenomas with high-grade dysplasia in the same colonic segment within one year after the baseline colonoscopy.

|  | **n (%)** | **No of person-years** | **No of CRCs** | **Incidence rate per 1000 person-years** | **Univariable HR (95% CI)** | ***P* value^a^** | **Multivariable HR (95% CI)** | ***P* value^a^** |
| --- | --- | --- | --- | --- | --- | --- | --- | --- |
| **Total** | 814(100%) | 2270.8 | 11 | 4.8 |  |  |  |  |
| **Sex** |  |  |  |  |  |  |  |  |
| women | 306(37.6%) | 830.8 | 5 | 6.0 | 1(ref) |  |  |  |
| men | 508(62.4%) | 1439.9 | 6 | 4.2 | 0.72(0.22-2.34) | 0.580 |  |  |
| **Age at baseline (years)** |  |  |  |  |  |  |  |  |
| <55 | 366(45.0%) | 1187.5 | 5 | 4.2 | 1(ref) |  |  |  |
| 55-64 | 242(29.7%) | 607.0 | 2 | 3.3 | 0.91(0.18-4.74) | 0.912 |  |  |
| 65-74 | 156(19.2%) | 366.9 | 3 | 8.2 | 2.28(0.54-9.69) | 0.264 |  |  |
| ≥75 | 50(6.1%) | 109.4 | 1 | 9.1 | 2.96(0.33-26.2) | 0.330 |  |  |
| **Location of adenomas with HGD**^b^ |  |  |  |  |  |  |  |  |
| Only distal | 619(76.0%) | 1705.5 | 7 | 4.1 | 1(ref) |  |  |  |
| Any proximal | 195(24.0%) | 565.2 | 4 | 7.1 | 1.73(0.51-5.91) | 0.382 |  |  |
| **Number of adenomas with HGD** |  |  |  |  |  |  |  |  |
| 1 | 730(89.7%) | 2015.0 | 8 | 4.0 | 1(ref) |  |  |  |
| ≥2 | 84(10.3%) | 255.8 | 3 | 11.7 | 3.04(0.81-11.49) | 0.101 |  |  |
| **Size of adenomas with HGD**^c^ |  |  |  |  |  |  |  |  |
| <10 mm | 109(13.4%) | 327.0 | 1 | 3.1 | 1(ref) |  |  |  |
| 10-19 mm | 306(37.6%) | 871.6 | 5 | 5.7 | 2.05(0.24-17.65) | 0.514 |  |  |
| ≥20 mm | 399(49.0%) | 1072.3 | 5 | 4.7 | 1.59(0.19-13.65) | 0.671 |  |  |
| **Synchronous PMPs**^d^ |  |  |  |  |  |  |  |  |
| No | 389(47.8%) | 1121.8 | 3 | 2.7 | 1(ref) |  |  |  |
| Low-risk polyps | 305(37.5%) | 790.8 | 5 | 6.3 | 2.52(0.60-10.55) | 0.207 |  |  |
| High-risk polyps | 120(14.7%) | 358.2 | 3 | 8.4 | 3.15(0.64-15.62) | 0.160 |  |  |
| **Resection** **range**^e^ |  |  |  |  |  |  |  |  |
| Segmental bowel resection | 114(14.0%) | 293.9 | 0 | 0 | 1(ref) |  |  |  |
| Local resection | 700(86.0%) | 1976.9 | 11 | 5.6 | 25.28(0.01-46890.64) | 0.400 |  |  |
| **Quality of baseline colonoscopy**^f^ |  |  |  |  |  |  |  |  |
| Adequate | 746(91.6%) | 2094.6 | 10 | 4.8 | 1(ref) |  |  |  |
| Inadequate | 68(8.4%) | 176.2 | 1 | 5.7 | 1.24(0.16-9.74) | 0.838 |  |  |
| **Baseline serum CEA level** |  |  |  |  |  |  |  |  |
| 0-4.9ng/mL | 557(68.4%) | 1478.5 | 5 | 3.4 | 1(ref) |  | 1(ref) |  |
| ≥5ng/mL | 44(5.4%) | 88.9 | 2 | 22.5 | 7.00(1.35-36.28) | 0.020 | 7.87(1.46-42.39) | 0.016 |
| NA | 213(26.2%) | 703.4 | 4 | 5.7 | 1.63(0.44-6.09) | 0.464 | 1.38(0.37-5.19) | 0.618 |
| **Number of surveillance visits** |  |  |  |  |  |  |  |  |
| 0 | 375(46.1%) | 558.0 | 7 | 12.5 | 1(ref) |  | 1(ref) |  |
| 1 | 197(24.2%) | 543.2 | 2 | 3.7 | 0.27(0.06-1.32) | 0.106 | 0.23(0.05-1.16) | 0.075 |
| ≥2 | 242(29.7%) | 1169.6 | 2 | 1.7 | 0.10(0.02-0.50) | 0.005 | 0.09(0.02-0.49) | 0.005 |

^a^*P* values were calculated with the likelihood ratio test. Differences with P<0.05 were considered statistically significant.

^b^Distal was defined as distal to the splenic flexure. Proximal was defined as proximal to the splenic flexure.

^c^Size was defined according to the largest adenomas with HGD seen at baseline.

^d^Low-risk polyps were defined as polyps <10mm in size and <5 in number and containing exclusively tubular/hyperplastic histology. High-risk polyps included tubulovillous adenomas, villous adenomas, sessile serrated polyps, traditional serrated adenomas, polyps ≥10mm in size and polyps ≥5 in number.

^e^Local resection included endoscopic resection (eg, cold/hot biopsy forceps, endoscopic snare polypectomy, endoscopic mucosal resection, endoscopic submucosal dissection) and transanal local excision.

^f^The baseline colonoscopy was inadequate if bowel cleansing was rated "poor" or "very poor", or if cecal intubation could not be achieved.

Abbreviations: CRC, colorectal cancer; HR, hazard ratio; CI, confidence interval; HGD, high-grade dysplasia; PMP, premalignant polyp; CEA, carcinoembryonic antigen; NA, not available.

Supplementary Table S9. Cumulative incidence of colorectal cancer, without excluding patients who developed colorectal cancers or adenomas with high-grade dysplasia in the same colonic segment within one year after the baseline colonoscopy.

|  | **n (%)** | **No of**  **person-years** | **No of CRCs** | **Incidence rate per 1000 person-years** | **5-year cumulative incidence (95% CI)^a^** | **10-year cumulative incidence (95% CI)^a^** | ***P* value^b^** |
| --- | --- | --- | --- | --- | --- | --- | --- |
| **Total** | 814(100%) | 2270.8 | 11 | 4.8 | 1.8% (0.8-4.0) | 5.7% (2.7-11.5) |  |
| **Sex** |  |  |  |  |  |  | 0.578 |
| women | 306(37.6%) | 830.8 | 5 | 6.0 | 2.8% (0.8-9.0) | 7.5% (0.3-19.1) |  |
| men | 508(62.4%) | 1439.9 | 6 | 4.2 | 1.3% (0.5-3.4) | 4.7% (1.6-13.5) |  |
| **Age at baseline (years)** |  |  |  |  |  |  | 0.200 |
| <55 | 366(45.0%) | 1187.5 | 5 | 4.2 | 1.6% (0.5-5.3) | 4.4% (1.6-11.5) |  |
| 55-64 | 242(29.7%) | 607.0 | 2 | 3.3 | 0.5% (0.1-3.6) | 4.2% (0.8-21.7) |  |
| 65-74 | 156(19.2%) | 366.9 | 3 | 8.2 | 3.2% (0.7-13.6) | － |  |
| ≥75 | 50(6.1%) | 109.4 | 1 | 9.1 | 5.6% (0.8-33.4) | － |  |
| **Location of adenomas with HGD**^c^ |  |  |  |  |  |  | 0.376 |
| Only distal | 619(76.0%) | 1705.5 | 7 | 4.1 | 1.6% (0.6-4.6) | 5.8% (2.4-13.6) |  |
| Any proximal | 195(24.0%) | 565.2 | 4 | 7.1 | 2.5% (0.8-7.9) | 5.5% (1.7-17.4) |  |
| **Number of adenomas with HGD** |  |  |  |  |  |  | 0.084 |
| 1 | 730(89.7%) | 2015.0 | 8 | 4.0 | 1.5% (0.6-4.0) | 5.0% (2.1-11.6) |  |
| ≥2 | 84(10.3%) | 255.8 | 3 | 11.7 | 4.2% (1.0-16.5) | － |  |
| **Size of adenomas with HGD**^d^ |  |  |  |  |  |  | 0.860 |
| <10 mm | 109(13.4%) | 327.0 | 1 | 3.1 | － | 6.7% (1.0-38.7) |  |
| 10-19 mm | 306(37.6%) | 871.6 | 5 | 5.7 | 2.1% (0.8-5.9) | － |  |
| ≥20 mm | 399(49.0%) | 1072.3 | 5 | 4.7 | 2.0% (0.6-6.7) | 5.6% (2.1-14.6) |  |
| **Synchronous PMPs**^e^ |  |  |  |  |  |  | 0.122 |
| No | 389(47.8%) | 1121.8 | 3 | 2.7 | 1.3% (0.3-5.7) | 3.5% (0.9-12.9) |  |
| Low-risk polyps | 305(37.5%) | 790.8 | 5 | 6.3 | 1.4% (0.5-4.3) | 6.0% (2.0-16.9) |  |
| High-risk polyps | 120(14.7%) | 358.2 | 3 | 8.4 | 3.8% (1.0-14.5) | － |  |
| **Resection** **range**^f^ |  |  |  |  |  |  | 0.188 |
| Segmental bowel resection | 114(14.0%) | 293.9 | 0 | 0 | － | － |  |
| Local resection | 700(86.0%) | 1976.9 | 11 | 5.6 | 2.1% (0.9-4.6) | 6.6% (3.2-13.6) |  |
| **Quality of baseline colonoscopy**^g^ |  |  |  |  |  |  | 0.837 |
| Adequate | 746(91.6%) | 2094.6 | 10 | 4.8 | 16% (0.7-4.0) | 5.7% (2.7-12.0) |  |
| Inadequate | 68(8.4%) | 176.2 | 1 | 5.7 | 3.6% (0.5-22.8) | － |  |
| **Baseline serum CEA level** |  |  |  |  |  |  | 0.032 |
| 0-4.9ng/mL | 557(68.4%) | 1478.5 | 5 | 3.4 | 0.8% (0.3-2.5) | － |  |
| ≥5ng/mL | 44(5.4%) | 88.9 | 2 | 22.5 | 9.8% (2.2-37.4) | － |  |
| NA | 213(26.2%) | 703.4 | 4 | 5.7 | 2.5% (0.6-9.8) | 9.9% (3.4-27.0) |  |
| **Number of surveillance visits** |  |  |  |  |  |  | 0.001 |
| 0 | 375(46.1%) | 558.0 | 7 | 12.5 | 6.9% (2.6-18.1) | － |  |
| 1 | 197(24.2%) | 543.2 | 2 | 3.7 | 1.7% (0.2-11.4) | － |  |
| ≥2 | 242(29.7%) | 1169.6 | 2 | 1.7 | － | 3.0% (0.7-12.2) |  |

^a^Cumulative incidence was estimated using the Kaplan-Meier method. Some categories of the specified variable could not be estimated.

^b^*P* values were calculated with the log-rank test to compare cumulative incidence among each category of the specified variable. Differences with P<0.05 were considered statistically significant.

^c^Distal was defined as distal to the splenic flexure. Proximal was defined as proximal to the splenic flexure.

^d^Size was defined according to the largest adenomas with HGD seen at baseline.

^e^Low-risk polyps were defined as polyps <10mm in size and <5 in number and containing exclusively tubular/hyperplastic histology. High-risk polyps included tubulovillous adenomas, villous adenomas, sessile serrated polyps, traditional serrated adenomas, polyps ≥10mm in size and polyps ≥5 in number.

^f^Local resection included endoscopic resection (eg, cold/hot biopsy forceps, endoscopic snare polypectomy, endoscopic mucosal resection, endoscopic submucosal dissection) and transanal local excision.

^g^The baseline colonoscopy was inadequate if bowel cleansing was rated "poor" or "very poor", or if cecal intubation could not be achieved.

Abbreviations: CRC, colorectal cancer; CI, confidence interval; HGD, high-grade dysplasia; PMP, premalignant polyp; CEA, carcinoembryonic antigen; NA, not available.
